# The Three-Dimensional Signal Collection Field for Fiber Photometry in Brain Tissue

**DOI:** 10.3389/fnins.2019.00082

**Published:** 2019-02-26

**Authors:** Marco Pisanello, Filippo Pisano, Minsuk Hyun, Emanuela Maglie, Antonio Balena, Massimo De Vittorio, Bernardo L. Sabatini, Ferruccio Pisanello

**Affiliations:** ^1^Istituto Italiano di Tecnologia, Center for Biomolecular Nanotechnologies, Lecce, Italy; ^2^Department of Neurobiology, Howard Hughes Medical Institute, Harvard Medical School, Boston, MA, United States; ^3^Dipartimento di Ingeneria dell'Innovazione, Università del Salento, Lecce, Italy

**Keywords:** fiber photometry, optogenetics, optical fibers, collection volumes, collection fields

## Abstract

Fiber photometry is used to monitor signals from fluorescent indicators in genetically-defined neural populations in behaving animals. Recently, fiber photometry has rapidly expanded and it now provides researchers with increasingly powerful means to record neural dynamics and neuromodulatory action. However, it is not clear how to select the optimal fiber optic given the constraints and goals of a particular experiment. Here, using combined confocal/2-photon microscope, we quantitatively characterize the fluorescence collection properties of various optical fibers in brain tissue. We show that the fiber size plays a major role in defining the volume of the optically sampled brain region, whereas numerical aperture impacts the total amount of collected signal and, marginally, the shape and size of the collection volume. We show that ~80% of the effective signal arises from 10^5^ to 10^6^ μm^3^ volume extending ~200 μm from the fiber facet for 200 μm core optical fibers. Together with analytical and ray tracing collection maps, our results reveal the light collection properties of different optical fibers in brain tissue, allowing for an accurate selection of the fibers for photometry and helping for a more precise interpretation of measurements in terms of sampled volume.

## Introduction

In the last decade, optogenetics has become widely used for optical control of neural activity (Miesenböck, 2009; Deisseroth, 2011; Häusser, 2014). Simultaneously, new implantable devices, mainly based on waveguides (Zorzos et al., 2012; Pisanello et al., 2014, 2017, 2018; Canales et al., 2015; Segev et al., 2016; Park et al., 2017; Pisano et al., 2018) or micro light emitting diodes (μLEDs) (Kim et al., 2013; McAlinden et al., 2013, 2015; Goßler et al., 2014; Wu et al., 2015; Scharf et al., 2016), have been developed for light delivery in the living brain. Recently these optical approaches have been extended to monitor neural circuits by detecting time-varying signals from diverse genetically-encoded fluorescent indicators of neural activity, neuromodulator action, and membrane potential (Fluhler et al., 1985; Loew, 1996; Miyawaki et al., 1997; Slovin et al., 2002; Petersen et al., 2003; Emiliani et al., 2015). While new devices utilizing integrated photodetectors and μLEDs have been described (Lu et al., 2018), traditional flat-cleaved optical fibers are broadly used for both triggering and collecting fluorescence *in vivo* in freely behaving animals. This approach is typically referred to as fiber photometry (Lütcke et al., 2010; Grienberger et al., 2012; Cui et al., 2013, 2014; Stroh et al., 2013; Adelsberger et al., 2014; Gunaydin et al., 2014; Chen et al., 2015; Fuhrmann et al., 2015; Kim et al., 2016; Matthews et al., 2016; Nieh et al., 2016; Lovett-Barron et al., 2017; Muir et al., 2017; Schwalm et al., 2017; Selimbeyoglu et al., 2017; He et al., 2018; Luo et al., 2018; Meng et al., 2018; Simone et al., 2018).

While the influence of optical fibers' constitutive parameters on emission properties and light delivery geometries in the brain are well-known (Aravanis et al., 2007; Yizhar et al., 2011; Schmid et al., 2016), similar information for fluorimetry performances is not yet available. Even though analytical models to estimate light collection field of optical fibers in quasi-transparent medium has been derived (Engelbrecht et al., 2009), the use of Monte Carlo simulations (Pfefer et al., 2001, 2002; Bargo et al., 2002, 2003a,b) or direct experimental measurements (Tai et al., 2007; Ryu et al., 2015) are required to properly assess spatial dependence of fluorimetry performances with high spatial resolution. Such information is necessary in order to select the optimal optical fibers to collect light from the brain region of interest as well as to interpret photometry measurements.

Here we evaluate the fluorescence collection properties of optical fibers typically employed in fiber photometry. We characterize the extension and shape of the probed volume and collected signal, evaluating the effects of fibers' constitutive parameters. Through a combined confocal/2-photon laser-scanning microscope we measured the light *collection* and *emission* fields in brain tissue whose combination determines the photometry efficiency field ρ(*x*,*y*,*z*) (Zhu and Yappert, 1992; Tai et al., 2007). These provide a quantitative estimation of collection volumes as a function of fiber numerical aperture (NA) and core diameter (*a*), together with an assessment of signal decay as a function of the position with respect to the fiber facet. Comparing data between different fibers, we found that NA has a secondary effect on photometry properties and that fiber core size is the chief parameter in defining the collection volume. Together with analytical and ray tracing collection maps, our data highlight aspects of light collection from brain tissue often overlooked in biological fiber photometry applications, with optical fibers having different NAs that have not been quantitatively compared yet.

## Results

### Numerical Estimation of Optical Fibers Collection Field

**Figure 1:**
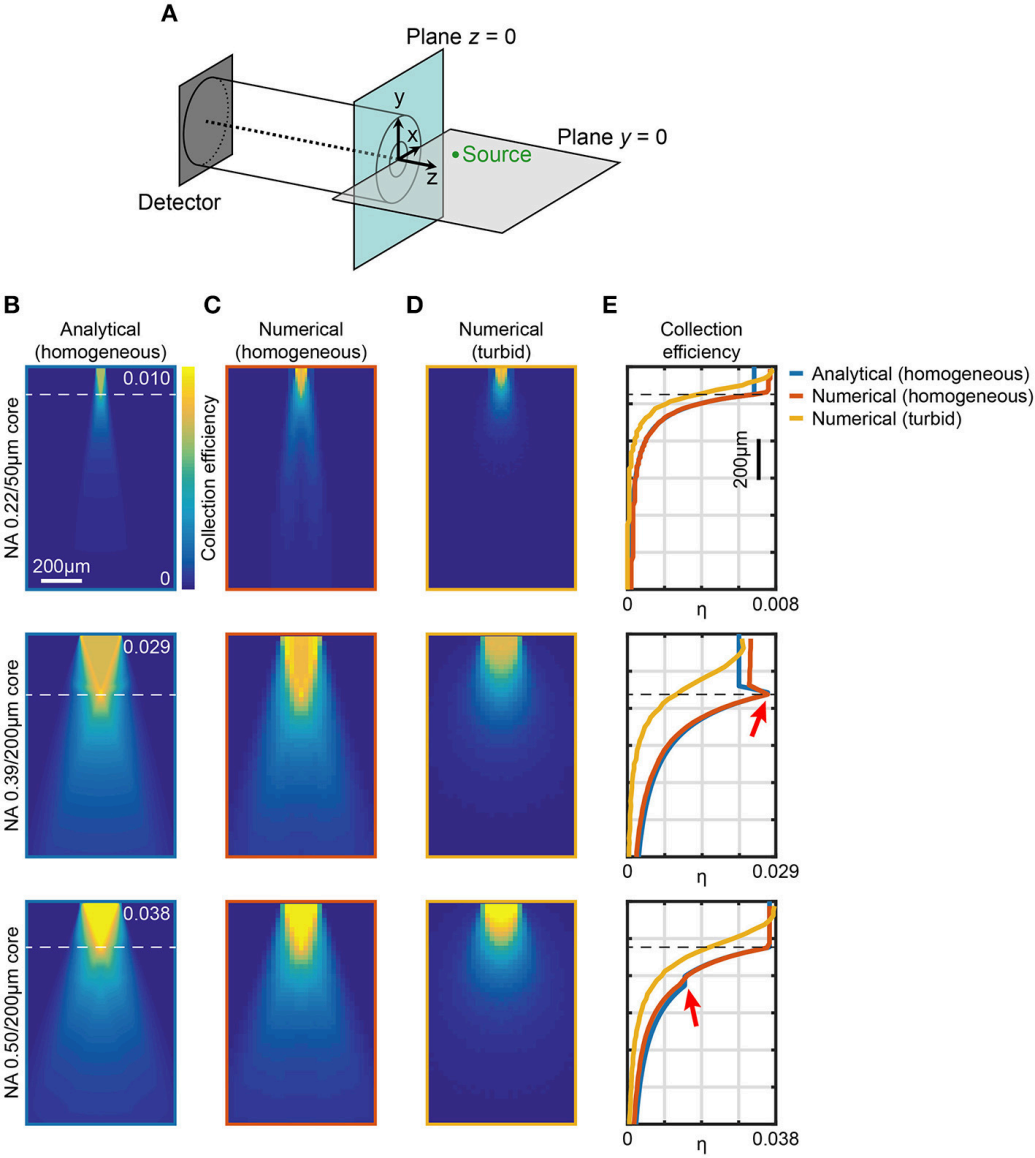
Computational models of light collection efficiency for optical fibers. **(A)** Reference system used throughout the manuscript. An example point source (green) is shown in the plane *y* = 0. **(B)** Analytical calculations of collection efficiency diagrams for light emitting from point sources locating in the *xz* (*y* = 0) plane for three different fibers. Data are shown for 0.22NA/50 μm, 0.39NA/200 μm, and 0.50NA/200 μm optical fibers, as indicated, immersed in a transparent homogeneous medium (*n* = 1.335). The horizontal dashed lines represent $z_{0}=a\cdot {\left[2\tan\left(\frac{\text{NA}}{n}\right)\right]}^{-1}$. **(C,D)** Ray tracing simulations of collection efficiency diagrams from a point source (λ = 520 nm) for same fibers as **(B)** immersed in a transparent homogeneous medium (*n* = 1.335) **(C)** or in a turbid medium (Henyey-Greenstein scattering, *n* = 1.360, *l* = 48.95 μm, *g* = 0.9254, *T* = 0.9989) **(D)**. **(E)** Comparison of axial collection efficiency (*x* = 0, *y* = 0) for 0.22NA/50 μm, 0.39NA/200 μm, and 0.50NA/200 μm optical fibers at λ = 520 nm immersed in a homogeneous medium (blue curve and orange curve for analytical and numerical data, respectively) and in a turbid medium (yellow curve). The red arrows indicate the effect of light collection through the cladding. The horizontal dashed lines represent *z*_0_.

As schematically represented in Figure 1A, the light generated from an isotropic fluorescent source is collected by an optical fiber with a certain efficiency that depends on the optical fiber's properties (numerical aperture and diameter) and on the properties of the medium between the source and the fiber (refractive index, absorption and scattering). For a given fiber with core diameter *a* and numerical aperture NA, immersed in a homogeneous medium with refractive index *n*, the analytical approach provided by Engelbrecht et al. (2009) estimates the 2D map of collection efficiency ψ(NA, *n, a, x, z*) as the fraction of the power collected by the fiber core from an isotropic point source located in the (*x, z*) plane (see Figure 1A for axis definition). To numerically estimate the collection field of optical fibers typically employed for *in vivo* fiber photometry (i.e., considering tissue absorption and scattering) we combined the approach in Engelbrecht et al. (2009) with a ray tracing model. In the following, we first extend the method proposed by Engelbrecht et al. (2009) to take into account the light entering the waveguide from the cladding front face; we then use the results to validate a ray tracing model that numerically estimates the fiber collection field in scattering brain tissue (see section Materials and Methods).

The collection efficiency η for a fiber with core diameter *a*, cladding diameter *b*, core refractive index *n*_core_, and cladding refractive index *n*_clad_ can be written as

(1) $$\begin{array}{l} \eta \left(\text{NA},{\text{n}}_{\text{core}},\;n,a,b,x,z\right)=\psi \left(\text{NA},\;n,a,x,z\right)\\ \;+\psi \left({\text{NA}}_{\text{eq}},\;n,b,x,z\right)-\psi ({\text{NA}}_{\text{eq}},\;n,a,x,z), \end{array}$$

where $\text{N}{\text{A}}_{\text{eq}}=\sqrt{n_{\text{clad}}^{2}-n^{2}}=\sqrt{n_{\text{core}}^{2}-{\text{NA}}^{2}-n^{2}}$ is the equivalent numerical aperture of the cladding/external medium interface (Snyder and Love, 1983), with the term ψ (NA_eq_, *n, b, x, z*) − ψ (NA_eq_, *n, a, x, z*) accounting for light collected by the cladding. Taking advantage of the axial symmetry of the system, values of η throughout the whole space filled by the external medium can be obtained. Meridional slices (*y* = 0) of such volumes are shown in Figure 1B for optical fibers with three different configurations of NA/core diameter commonly used for fiber photometry experiments (0.22/50 μm, 0.39/200 μm, and 0.50/200 μm, respectively), for a homogeneous medium with *n* = 1.335. These maps show the presence of a region with constant collection efficiency next to the fiber core, roughly described by a cone with base coincident with the fiber facet surface and vertex lying on the waveguide axis at *z*_0_ (Engelbrecht et al., 2009), where

(2) $$z_{0}=\frac{a}{2\cdot \text{tan}\left[{\text{sin}}^{-1}\left(\frac{\text{NA}}{n}\right)\right]}\approx \frac{a}{2\cdot \text{tan}\left(\frac{\text{NA}}{n}\right)}.$$

Interestingly, for 0.39/200 μm fiber the maximum collection efficiency region lies along the lateral surface of the cone, due to the fact that NA_eq_ > NA, whereas this does not happen in the case of the 0.50/200 μm fiber for which NA_eq_ < NA. This difference between 0.39/200 μm and 0.50/200 μm fibers is clearly visible also in the axial collection profiles (blue lines in Figure 1E). In the case of the 0.39/200 μm fiber, as a result of cladding collection, a peak in the collection efficiency is observed at the boundary of the constant region close to the fiber (red arrow in Figure 1E, middle panel). In contrast, for the 0.50/200 μm fiber cladding collection leads only to a small plateau indicated by the red arrow in the bottom panel of Figure 1E. The influence of cladding on collected signal for different parameters is summarized in Supplementary Figure 1, showing the comparison between the volumes enclosed by iso-surfaces at several values of η for fibers with NA = 0.22, 0.39, 0.50, 0.66, and core cladding/diameters *a*/*b* = 200 μm/225 μm, 400 μm/425 μm. Cladding contribution leads to a general increase of the collection volume, more pronounced for fibers where NA_eq_ > NA: for the 0.39 NA fiber considered in this work, the cladding generates ~57% and ~27% volume increase for 200 and 400 μm core, respectively. Performances of fibers with higher *a*/*b* ratio or with NA_eq_ < NA are less affected by the cladding collection.

Ray-tracing simulations were performed by scanning an isotropic point source at λ = 520 nm across the *xz* plane (the ray tracing setup is shown in Supplementary Figure 2). Modeled light rays entering the fiber within NA_eq_ were propagated through a short length of patch fiber (10 mm) and registered if they reached a hypothetical detector at the distal end of the fiber. Results for core/cladding fibers are displayed in Figure 1C. This configuration simulated the potential leakage of light rays outside NA_eq_ that propagate in the cladding. A comparison in terms of axial collection profiles (Figure 1E) shows a very good agreement with the analytical model for both the geometrical behavior and the absolute collection efficiency values. In particular, the maximum collection efficiency η for the 0.39/200 μm fiber was found to be ~ 0.03 whereas in the case 0.50/200 μm it was estimated to be ~0.04. The model matches well also with the analytical approach of Engelbrecht et al. (2009) when cladding collection is neglected (inset of Supplementary Figure 2).

Since the ray-tracing approach and the analytical method gave consistent results, we extended the numerical simulations to model turbid media, such as scattering brain tissue. We modeled the medium around the fiber with a Henyey-Greenstein scattering function to simulate absorption and scattering properties of brain tissue (Zinter and Levene, 2011; Yona et al., 2016) (refractive index *n* = 1.360, mean free path *l* = 48.95 μm, anisotropy parameter *g* = 0.9254, transmission coefficient *T* = 0.9989). The resulting maps of collection efficiency for 0.22/50 μm, 0.39/200 μm, and 0.50/200 μm fibers are shown in Figure 1D. As a comparison, the axial profiles of analytical and numerical estimation of η for both transparent and turbid media are reported in Figure 1E for all the investigated fibers. When absorption and scattering of the medium are considered, the constant region in collection efficiency almost disappears, and η starts decreasing immediately after the fiber facet. The maximum η value remains ~ 65% higher for the 0.50/200 μm fiber with respect to 0.39/200 μm. However, the collection efficiency decrease is slightly steeper for the 0.50/200 μm fiber, reaching 50% of the maximum at 250 μm from the fiber facet, compared to 300 μm observed for the 0.39/200 μm fiber.

### Empirical Model for Collection Volumes

**Figure 2:**
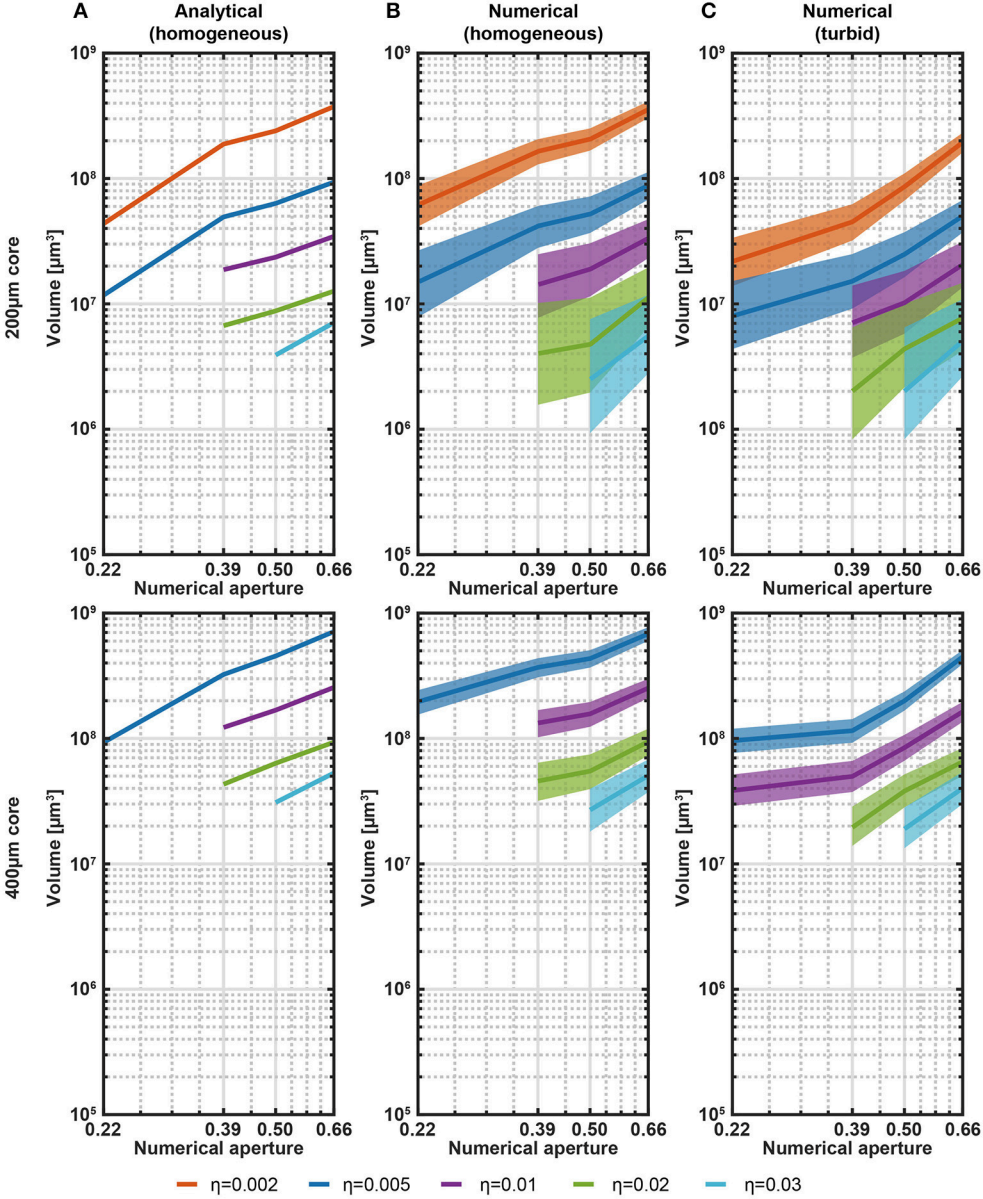
Analytical and numerical estimation of collection volumes. **(A)** Volume iso-surfaces at different η estimated with the analytical model in transparent medium as a function of NA. Data are shown for η = 0.002, 0.005, 0.01, 0.02, 0.03 and for fibers with core/cladding diameters *a*/*b* = 200 μm/225 μm, 400 μm/425 μm (top and bottom panels, respectively). Missing data at low NA means null volume. **(B)** Same analysis of **(A)** shown for the ray-tracing model in transparent medium. The width of the curves represents the error in the volume estimation introduced by the domain discretization. **(C)** Same analysis of **(B)** shown for the ray-tracing model in a turbid medium to simulate brain tissue. Scattering was modeled with Henyey-Greenstein formulation (*n* = 1.360, *l* = 48.95 μm, *g* = 0.9254, *T* = 0.9989). The width of the curves represents the error in the volume estimation introduced by the domain discretization.

Collection fields returned from both analytical model and ray-tracing simulations were used to obtain an empirical model of the probed volume as a function of fiber NA and size, assuming axially symmetric distribution, in quasi-transparent medium and brain tissue. Volumetric data for different values of η for fibers with NA = 0.22, 0.39, 0.50, 0.66, and *a*/*b* = 200 μm/225 μm, 400 μm/425 μm are reported in Figures 2A,B for the analytical and the ray tracing models, respectively. These plots highlight that core size plays an important role in defining the volume from which light is gathered, with increased NA impacting for a lower amount in the volume increase.

On the base of the good match between the empirical and ray-tracing models, this latter is used to estimate the collection volumes also in turbid medium, as shown in Figure 2C. The overall behavior is the same observed in quasi-transparent medium, with collection volumes in turbid medium being ~2 times smaller than volumes in transparent medium. Even though Figure 2C covers common NAs and core/cladding sizes, a rough estimation of the collected volumes from fibers with parameters in between the data points can be interpolated from the plots.

### Direct Measurement of Collection Field in Quasi-Transparent Fluorescent Media

**Figure 3:**
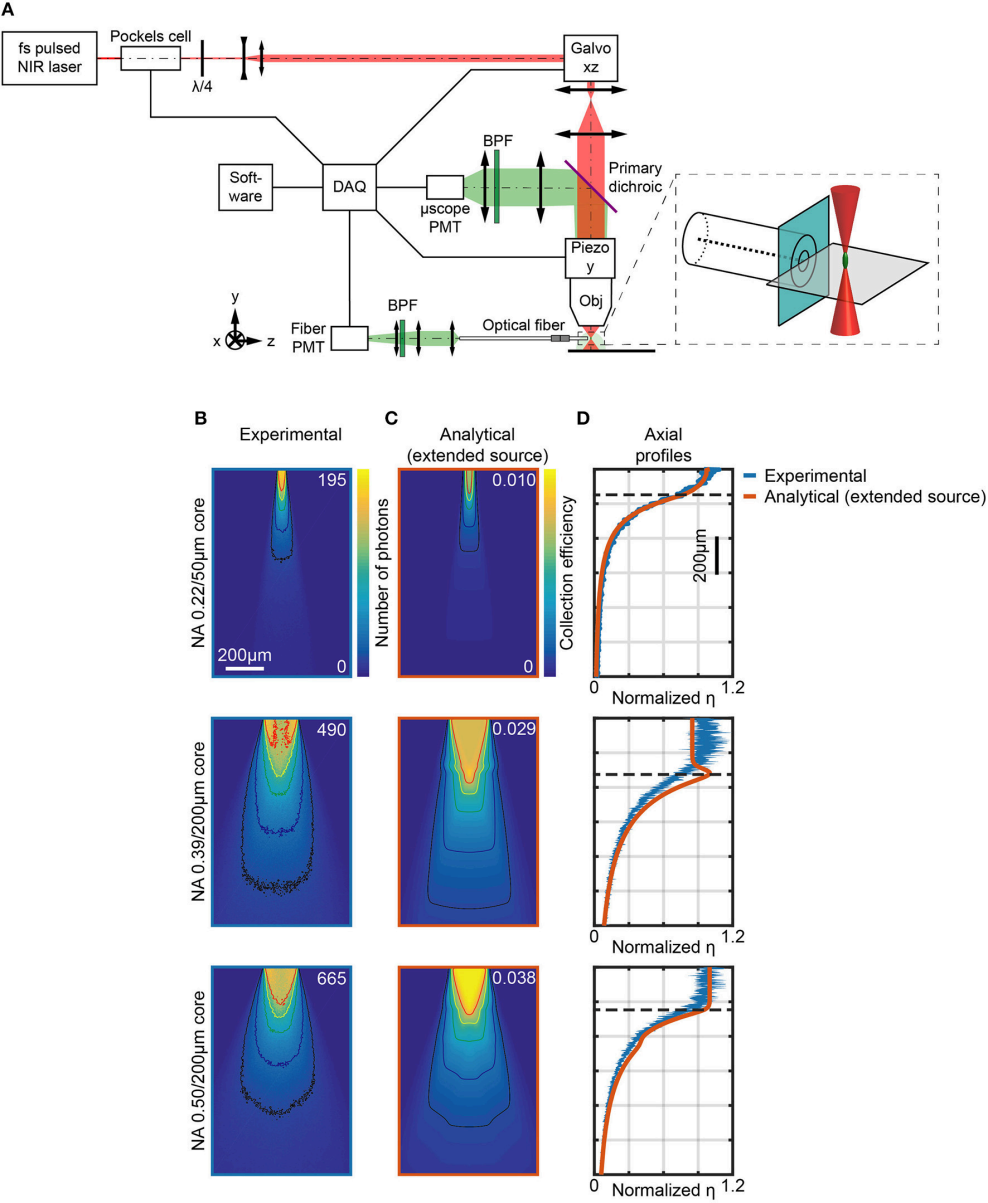
Measurements of light collection efficiency using 2-photon generated fluorescent point sources. **(A)** Schematic representation of the two-photon microscope used to measure the collection field of optical fibers in quasi-transparent fluorescent medium. The inset shows a magnification of the fiber facet surroundings. **(B)** Section *y* = 0 of the collection field of 0.22/50 μm, 0.39/200 μm, and 0.50/200 μm optical fibers, as indicated, in a 30 μM PBS:fluorescein solution, obtained through the *fiber PMT* as shown in **(A)**. Isolines at 10%, 20%, 40%, 60%, and 80% of the maximum number of photons are shown (in black, blue, green, yellow, and red, respectively). **(C)** Analytical calculations of collection efficiency diagrams for the three fibers in **(B)** immersed in a transparent homogeneous medium (*n* = 1.335) assuming a gaussian source with lateral FWHM *r*_*x, z*_ = 3 μm, axial FWHM *r*_*y*_ = 32 μm. Isolines at 10%, 20%, 40%, 60%, and 80% of the maximum number of photons are shown (in black, blue, green, yellow, and red, respectively). **(D)** Comparison of normalized experimentally-measured (blue curve) and analytically-calculated (orange curve) axial collection efficiency profiles (*x* = 0, *y* = 0) for the same fibers in **(B)**. Normalization is done with respect to the average of the data points within the firsts 80 μm. The horizontal dashed lines represent *z*_0_. The width of the blue curves for the 0.39 NA/200 μm and 0.50 NA/200 μm fibers represents mean ∓ standard deviation over four different fibers.

A two-photon (2P) laser scanning system has been designed and built to directly measure the light collection field of optical fibers, in a configuration similar to Tai et al. (2007). A block diagram of the optical path is illustrated in Figure 3A: the optical fiber was submerged in a fluorescent PBS:fluorescein solution (30 μM) and a fs-pulsed near-infrared laser (λ_ex_ = 920 nm) was used to generate a fluorescent voxel that was scanned in three dimensions close to the fiber facet. Scan in the *xz* plane was obtained by a two-axis galvanometric scanhead on a ~1.4 × 1.4 mm^2^ field of view (FOV), with the microscope objective (Olympus XLFluor 4x/340 NA 0.28) mounted on a *y*-axis piezo focuser to obtain a volumetric scan. The voxel emission was collected by the same objective and detected by a non-descanned photomultiplier tube (“μ*scope PMT*”). This gave a measurement of the total fluorescence generated and, if needed, can be used to compensate for changes in excitation efficiency of the scanning point source. Simultaneously, the fraction of the voxel's fluorescence that was collected by the optical fiber and guided to a second PMT (“*fiber PMT*”) was measured. The point spread function (PSF) of the two-photon epifluorescence system was measured to be 3 μm laterally and 32 μm axially (see Supplementary Figure 3 and section Materials and Methods for details).

During volumetric raster scanning of the 2P spot, Scanimage software (Vidrio Technologies) was used to reconstruct images from both the μ*scope PMT* and the *fiber PMT* signals, allowing for a point-by-point mapping of the light intensity collected from the optical fiber within the scanned volume. Figure 3B shows the signal collected by the *fiber PMT* when the excitation was scanned across the *y* = 0 plane for three different type of optical fiber: 0.22/50 μm (Thorlabs FG050UGA, top panel), 0.39/200 μm (Thorlabs FT200UMT, middle panel), and 0.50/200 μm (Thorlabs FP200URT, bottom panel), with overlay of the isolines at 10%, 20%, 40%, 60%, and 80% of the maximum number of photons collected. Supplementary Figure 4 shows the signal collected along the *x* = 0 plane. A typical full volumetric scan is shown in the video of Supplementary Video 1, obtained with a 0.50/200 μm fiber. These images were corrected for unevenness of illumination within the FOV by using the related signal on the μ*scope PMT* (see Supplementary Figure 5). In addition, the gain *G* of the system was estimated by noise analysis at each measurement session and used to convert the PMTs signals into numbers of photons (see Materials and Methods for details). A direct measurement of η as ratio between the collected photons (the signal read by the *fiber PMT*) and the photons emitted by the fluorescent source (the signal read by the μ*scope PMT* corrected by the solid angle of collection of the microscope objective) can also be obtained. For a direct comparison, analytical collection maps were also computed convolving η and a three-dimensional function modeling the experimental PSF (Figure 3C) (details on this calculation are reported in Materials and Methods). The related axial profiles, shown normalized to the to the average of the points within the firsts 80 μm in Figure 3D, indicate good agreement between numerical predictions and the experimental data. Both analytical and experimental results show a difference between 0.39/200 μm and 0.50/200 μm fibers (Figures 3B–D). For 0.39/200 μm fibers, the region of maximum collection does not lie on the optical axis, but in two lobes near the boundary of the core: this can be ascribed to the fact that light is efficiently guided not only by the core-cladding interface, but also by the waveguide formed by the cladding and the external medium (i.e., the PBS:fluorescein solution).

**Figure 4:**
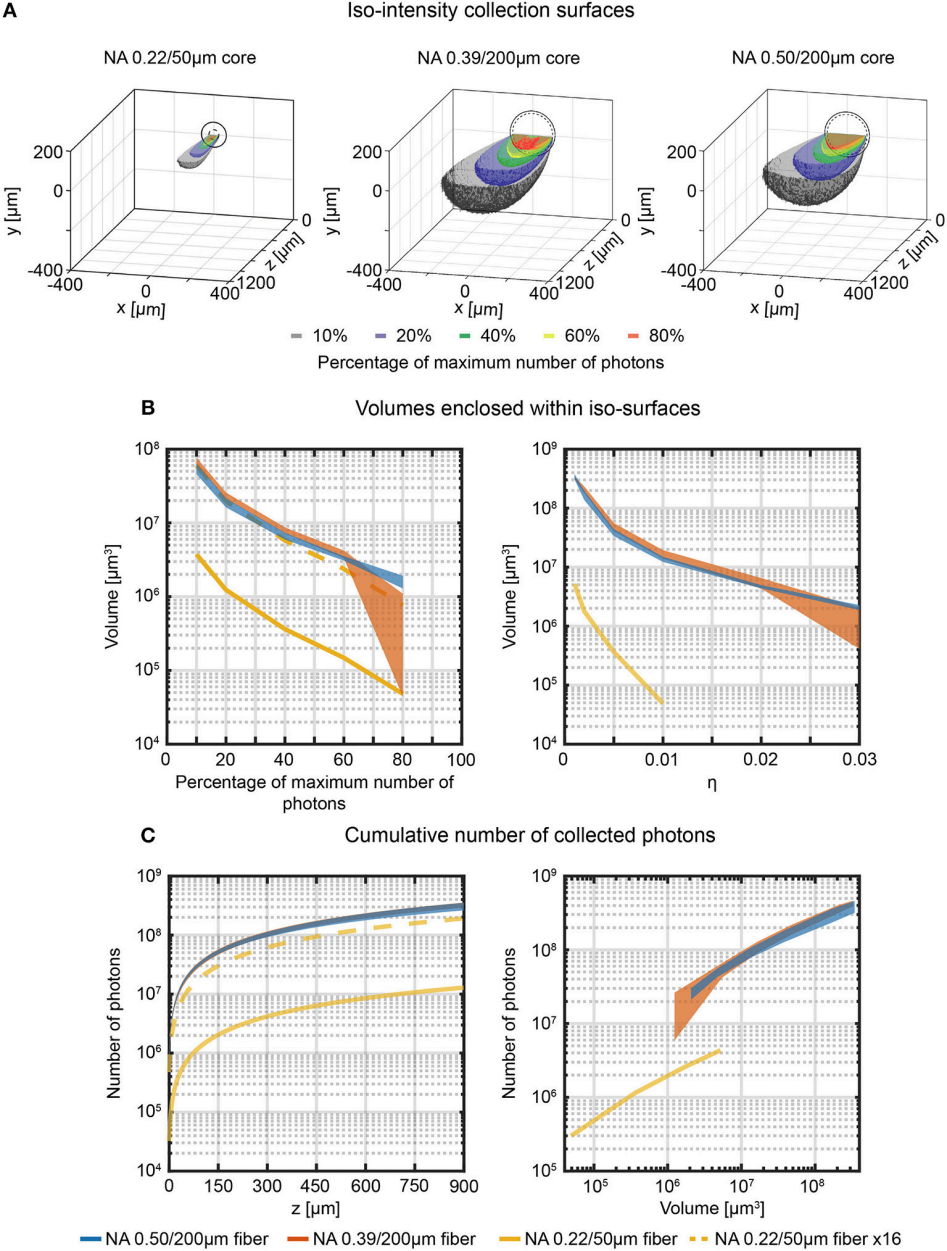
Effective light collection volumes in quasi-transparent medium. **(A)** Cross-sectional views of the 3-dimensional reconstructions of the collection field for 0.22/50 μm, 0.39/200 μm, and 0.50/200 μm fibers in quasi-transparent solution. Iso-intensity surfaces defining the boundaries at which the number of collected photons falls to 10%, 20%, 40%, 60%, and 80% of its maximum are shown (in black, blue, green, yellow and red, respectively). The continuous and dashed circles in the *xy* plane represent the cladding and the core boundaries, respectively. **(B)** Volumes enclosed by the iso-intensity surfaces at 10%, 20%, 40%, 60%, and 80% of the maximum number of photons (left panel) and at η = 0.001, 0.002, 0.005, 0.01, 0.02, 0.03 (right panel) for 0.22/50 μm, 0.39/200 μm, and 0.50/200 μm fibers (yellow, orange, and blue curves, respectively). The dashed yellow curve represents the data for the 0.22/50 μm fiber multiplied by a factor 16 to adjust for the smaller cross-sectional area of this fiber. The width of the curves for the 0.39NA/200 μm and 0.50NA/200 μm fibers represents mean ∓ standard deviation over four different fibers. **(C)** Cumulative number of photons collected by the three fibers as a function of the distance from the fiber facet (left panel, number of photons are shown in a volume 900 μm × 600 μm × *z*) and as a function of the volume enclosed within the iso-surfaces at fixed η (right panel). The dashed yellow curve represents the data points relative to the 0.22/50 μm fiber multiplied by a factor 16. The width of the curves for the 0.39NA/200 μm and 0.50NA/200 μm fibers represents mean ∓ standard deviation over four different fibers.

By assembling the data collected from adjacent *xz*-planes in the sampled volume, the full three-dimensional collection fields can be reconstructed and used to illustrate the iso-intensity surfaces at 10%, 20%, 40%, 60%, and 80% of the maximum number of collected photons (Figure 4A). The volumes enclosed by these surfaces reflect those from which a given fraction of the collected photons arise and hence determine the effective volume from which functional signals can be detected (Figure 4B, left). Collection volumes of 0.39NA/200 μm and 0.50NA/200 μm fibers behave very similar for relative intensities ≤60%. The 0.22NA/50 μm (which has a 16 times smaller core surface) shows consistently lower collection volumes. When the volumetric data for the 0.22NA/50 μm fiber are multiplied by a factor 16 (dotted yellow line in Figure 4B, left), collection volumes are close to those of 0.39/200 μm and 0.50/200 μm fibers. The same consideration can be done by evaluating collected photons within the iso-intensity surfaces at fixed value of absolute collection efficiency η (Figure 4B, right): 0.39NA/200 μm and 0.50NA/200 μm fibers behave similarly, with the 0.50NA/200 μm fiber providing lower variation among different fibers at higher collection efficiency. As users of fiber photometry are interested in the overall signal collected from a certain depth or from a certain volume, these parameters have been extracted from the volumetric collection fields. The absolute cumulative number of collected photons as a function of collection depth is shown in Figure 4C, left, while the photons flux emerging from a specific volume (defined within iso-intensity surfaces at fixed value of η) is displayed in Figure 4C (right). These data highlight that in quasi-transparent media these two figures of merit behave very similarly for 0.39NA/200 μm and 0.50NA/200 μm fibers (the ratio of the average number of photons collected throughout the range *z* = 0 μm−900 μm is ~1, as shown in Supplementary Figure 6A), while 0.22NA/50 μm fiber collects less photons from smaller volumes.

### Direct Measurement of Collection Field in Brain Slices

**Figure 5:**
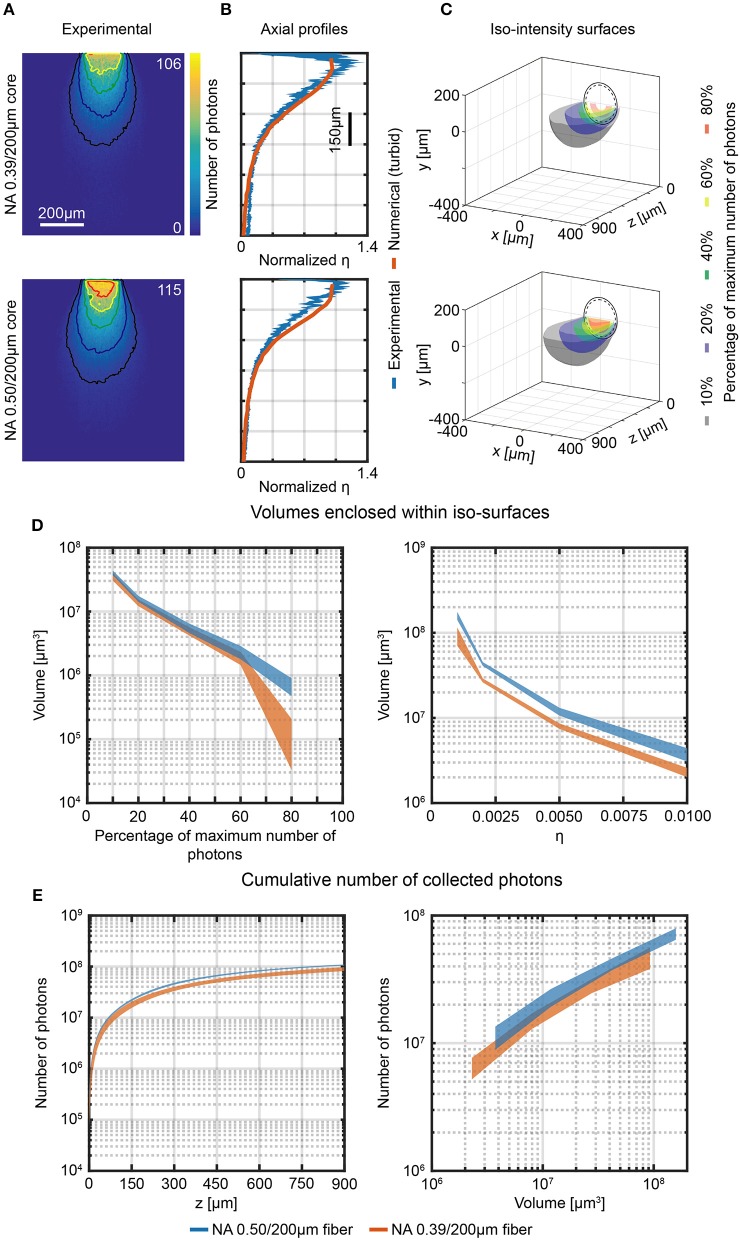
Photon collection efficiency and effective collective volumes in brain slice. **(A)** Section *y* = 0 of the collection field of 0.39/200 μm and 0.50/200 μm optical fibers, measured in a 300 μm thick fluorescently stained brain slice using the 2-photon scanning system shown in Figure 3. Isolines at 10%, 20%, 40%, 60%, and 80% of the maximum number of photons are shown (in black, blue, green, yellow and red, respectively). **(B)** Comparison of normalized axial profiles (*x* = 0, *y* = 0) of experimental (in brain slices, blue curves) and numerical data (in turbid medium, orange curves) for 0.39/200 μm and 0.50/200 μm optical fibers. Normalization is done with respect to the average of the data points within the firsts 80 μm. The width of the blue curves represents mean ∓ standard deviation over four different fibers. **(C)** Cross-sections of the 3-dimensional reconstruction of the collection field of 0.39/200 μm and 0.50/200 μm fibers. Iso-intensity surfaces defining the boundaries at which the number of collected photons falls to 10%, 20%, 40%, 60%, and 80% of its maximum are shown (in black, blue, green, yellow, and red, respectively). The continuous and dashed circles represent the cladding and the core boundaries, respectively. **(D)** Volumes enclosed by the iso-intensity surfaces at 10%, 20%, 40%, 60%, and 80% of the maximum number of photons (left panel) and at η = 0.001, 0.002, 0.005, 0.01 (right panel) for 0.39/200 μm and 0.50/200 μm fibers (orange, and blue curves, respectively). The width of the curves represents mean ∓ standard deviation over three different fibers. **(E)** Cumulative number of photons collected by 0.39/200 μm and 0.50/200 μm fibers as a function of the distance from the fiber facet (left panel, number of photons are shown in a volume 900 μm × 600 μm × *z*) and as a function of the volume enclosed within the iso-surfaces at fixed η (right panel). The width of the curves represents mean ∓ standard deviation over three different fibers.

The system depicted in Figure 3A was also used to measure the light collection field of 0.39/200 μm and 0.50/200 μm optical fibers in 300 μm thick brain slices stained with fluorescein. This was done to estimate the influence of tissue absorption and scattering on the geometrical features of light collection. Figure 5A shows the results of these measurements for the two investigated fibers on the plane *y* = 0, with overlay of the isolines at 10%, 20%, 40%, 60%, and 80% of the maximum signal detected from the *fiber PMT*. A clear difference compared to the measurement in PBS:fluorescein solutions is that the flat collection efficiency region was disrupted by tissue scattering so that it was not possible to define the characteristic point at *z*_0_ for either fibers. This was also seen in the axial collection profiles reported normalized to the average of the points within the firsts 80 μm in Figure 5B, which show a steep decrease of the collection curve starting at the fiber face. These findings were confirmed by comparing the results to those obtained using the ray-tracing model discussed above for both axial collection profiles (orange lines in Figure 5B) and their derivatives (Supplementary Figure 7A). It is also important to mention that, although collection diagrams in quasi-transparent media are fully symmetric (Supplementary Figure 8), the data in tissue present a certain degree of asymmetry due to the tissue conformation, which could also induce slightly uneven staining.

The *y* = 0, *x* < 0 half-planes of the measurements in Figure 5A were used to reconstruct the collection volume. The collection volume was reconstructed by 360° rotation after applying a 11 × 11 pixel moving average filter (details on the procedure are given in Materials and Methods). Iso-intensity surfaces of the reconstructed 3D collection field at 10%, 20%, 40%, 60%, and 80% of the maximum PMT counts value were calculated (Figure 5C), and collection volumes at the same relative threshold and for fixed value of η were determined (Figure 5D, left and right, panel, respectively). As expected, the collection volumes in tissue are smaller with respect to the volumes in fluorescent solution due to light absorption and scattering. In addition, the 0.50/200 μm fiber increased the average collection volume by a factor ~1.6 compared to the average volume collected by the 0.39/200 μm fiber (see Supplementary Figure 7B for a detailed plot of volumes ratio between 0.50 and 0.39 fibers for each η iso-intensity curve). The absolute value of collected photons as a function of collection depth and as a function of volume within iso-efficiency collection surfaces at fixed value of η are displayed in Figure 5E. 0.50NA/200 μm fiber collect slightly more photons than the 0.39NA/200 μm fiber (the ratio of the average number of photons collected throughout the range *z* = 0 μm−900 μm is ~1.24, matching with a 3% tolerance the ratio of 1.28 between the NAs, as shown in Supplementary Figure 6B).

### Photometry Efficiency in Brain Tissue

**Figure 6:**
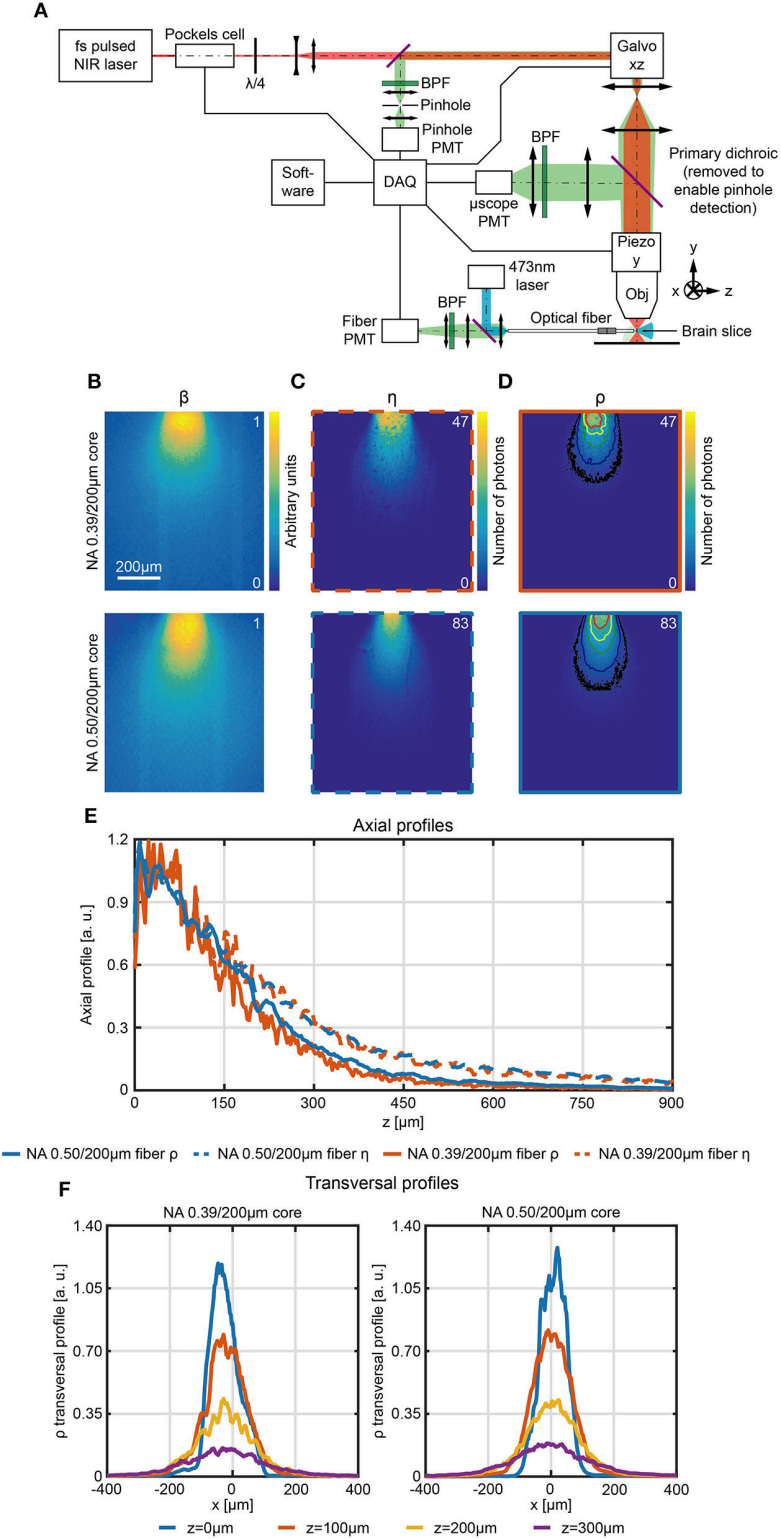
Scanning pinhole detection of the excitation light field for fiber optics. **(A)** Schematic representation of the optical path used to measure the photometry efficiency diagram of optical fibers in fluorescently stained brain slices. **(B–D)** Section *y* = 0 of the normalized light emission diagram β **(B)**, the collection efficiency η **(C)** and the photometry efficiency ρ **(D)** of 0.39/200 μm and 0.50/200 μm optical fibers, as indicated, measured in a 300 μm thick fluorescently stained brain slice. In **(D)** isolines at 10%, 20%, 40%, 60%, and 80% of the maximum efficiency are shown (in black, blue, green, yellow, and red, respectively). **(E)** Comparison of normalized axial profiles (*x* = 0, *y* = 0) between 0.39/200 μm and 0.50/200 μm fibers for photometry efficiency (orange and blue continuous curve, respectively) and collection efficiency (orange and blue dashed curve, respectively). Normalization is done with respect to the average of the points within the firsts 80 μm. **(F)** Normalized photometry efficiency transversal profiles at different depths (*z* = 0 μm, 100 μm, 200 μm, 300 μm, *y* = 0) for 0.39NA/200 μm and 0.50NA/200 μm fibers (left and right panel, respectively).

We described the approach used to estimate the collection efficiency of an optical fiber by scanning a point like source in the proximity of the fiber facet. However, in fiber photometry experiments fluorescence is generated by delivering excitation light (typically at 473 nm or 488 nm) and collecting the generated fluorescence (usually in the range 500 nm−550 nm) through the same fiber. Therefore, light intensity obtained from a specific position depends not only on how photons are collected from that point, but also on the efficiency at which fluorescence is excited at that point. A *photometry efficiency* parameter ρ can therefore be defined as:

(3) ρ(x,y,z)= η (x,y,z) · β(x,y,z)

where η is the collection efficiency and β is the normalized light emission diagram of the same optical fiber used to collect light (Zhu and Yappert, 1992; Tai et al., 2007). In brain tissue, η can be estimated with the 2P scanning method detailed in paragraph Direct Measurement of Collection Field In Quasi-Transparent Fluorescent Media. To estimate β in the same location where η is measured, a pinhole detection system was implemented in a de-scanned collection path (Figure 6A). The scanning pinhole allows light to reach the detector only if it arises from a conjugate location in the tissue; thus, its intensity is determined by the efficiency at which light reaches the point from the fiber. In this way fiber emission diagram is measured in brain slices uniformly stained with fluorescein. A block diagram of the experimental setup is shown in Figure 6A. A 473 nm CW laser source was coupled to the fiber distal end and provided the excitation light. The fluorescence light excited in the brain slice by the optical fiber was imaged on the galvanometric mirrors and scanned through a pinhole aperture that conveyed it on a *pinhole PMT*. This detector was used to reconstruct the fluorescence intensity map within the same FOV (same magnification and position) of the 2P scan used to estimate η (Figures 6B,C, respectively). A comparison of η and β in terms of axial decay is shown in Supplementary Figure 9. Upon normalization, β maps give an estimation of the light emission diagram and can be used in conjunction with the collection fields to estimate ρ (pixel by pixel product of η and β).

The resulting maps (Figure 6D, with overlay of the isolines at 10%, 20%, 40%, 60%, and 80% of the maximum photometry efficiency) contain in each pixel a value proportional to (i) the amount of fluorescence light excited by the fiber in that pixel and (ii) to the amount of light collected from that pixel. These values describe the relative contribution of signal arising from each voxel and thus determine the spatial distribution of the sources of signal collected during a fiber photometry recording. This analysis reveals that, as expected from the effect of tissue scattering, the axial profile of ρ falls off faster with distance from the fiber face than the collection only diagram (Figure 6E, profiles are normalized to the to the average of the points within the firsts 80 μm) for both the 0.39/200 μm and 0.50/200 μm fibers. Transversal profile of ρ along *x* at *z* = 0 μm, 100 μm, 200 μm, 300 μm for both fibers are shown in Figure 6F.

**Figure 7:**
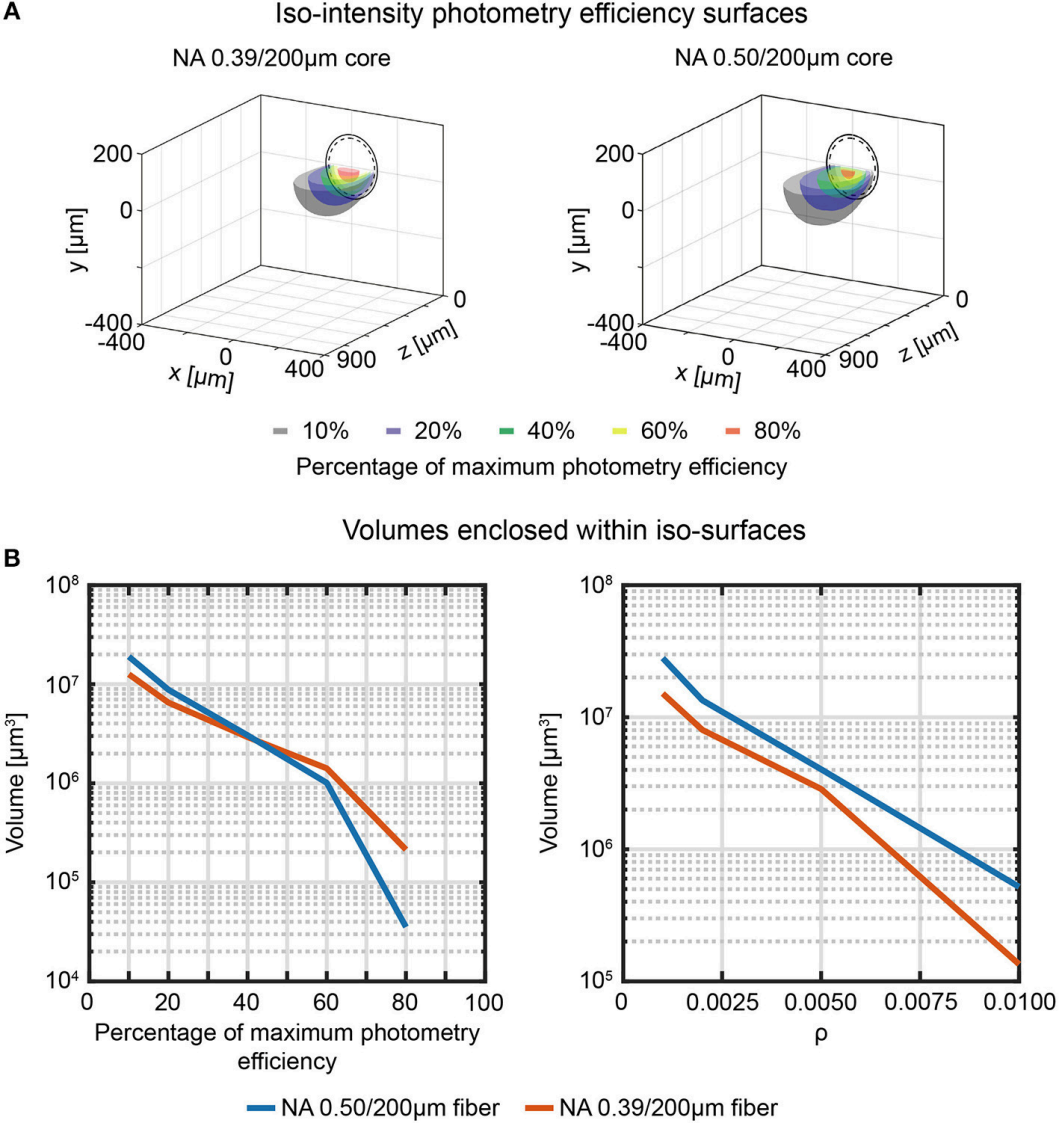
Effective fiber photometry sampling volumes in tissue. **(A)** Cross-sections of the 3-dimensional reconstruction of the photometry efficiency diagram of 0.39/200 μm and 0.50/200 μm fibers (left and right, respectively). Iso-intensity surfaces at 10%, 20%, 40%, 60%, and 80% efficiency are shown (in black, blue, green, yellow, and red, respectively). The continuous and dashed circles represent the cladding and the core boundaries, respectively. **(B)** Volumes enclosed by the iso-intensity surfaces at 10%, 20%, 40%, 60%, and 80% of the maximum photometry efficiency (left) and at ρ = 0.001, 0.002, 0.005, 0.01 (right) for 0.39/200 μm and 0.50/200 μm fibers (orange and blue curves, respectively).

Similarly, volumetric analysis analogous to the ones described in section Direct Measurement of Collection Field in Quasi-Transparent Fluorescent Media can be extended to photometry efficiency (Figure 7A) to determine the volumes enclosed by the iso-intensity surfaces at 10%, 20%, 40%, 60%, and 80% of the maximum photometry efficiency and at fixed values of ρ (Figure 7B left and right panel, respectively). The higher numerical aperture 0.50/200 μm fiber results in a collection volume ~2.2 times higher than the 0.39/200 μm fiber (on average: the plot of the ratio between the two datasets for the different ρ iso-intensity surfaces is reported in Supplementary Figure 10).

## Discussion and conclusion

Although fiber photometry is regularly employed to investigate the relationship between neural activity and behavior as well as connectivity in neural circuits (Lütcke et al., 2010; Grienberger et al., 2012; Cui et al., 2013, 2014; Stroh et al., 2013; Adelsberger et al., 2014; Gunaydin et al., 2014; Chen et al., 2015; Fuhrmann et al., 2015; Kim et al., 2016; Matthews et al., 2016; Nieh et al., 2016; Lovett-Barron et al., 2017; Muir et al., 2017; Schwalm et al., 2017; Selimbeyoglu et al., 2017; He et al., 2018; Luo et al., 2018; Meng et al., 2018; Simone et al., 2018), the fluorimetry properties of optical fibers inserted into the brain have not been well-characterized yet. In this work we evaluated the illumination and collection fields for widely-used multimode optical fibers in brain tissue, quantitatively estimating signal collection efficiency as well as size and shape of collection volumes.

This was achieved by implementing a combined confocal/two-photon laser-scanning microscope to measure both the collection and emission diagrams from the same fiber in the same region (Figures 3, 6) (Tai et al., 2007). The 2P path was used to estimate collection volumes in both quasi-transparent media and brain slices (Figures 4, 5, respectively), showing that collection volumes for 0.39 NA and 0.50 NA fibers with 200 μm core are almost the same in fluorescein and only slightly higher for 0.50 NA fibers in brain slices. The geometric distribution of the collection volume consistently agrees with analytical and numerical estimations based on theory and ray tracing models. The latter can therefore be confidently used to estimate the spatial dependence of light collection intensity once the scattering and absorption parameters in the Henyey-Greenstein model are known in terms of mean free path, anisotropy parameter, and transmission coefficient (all scripts are provided in Supplementary Material).

The observations on collection fields measured for 0.39 NA and 0.50 NA hold true also for the photometry efficiency fields ρ(*x*,*y*,*z*). According to our data, 0.39/200 μm and 0.50/200 μm fibers behave very similarly in terms of on-axis spatial decay of ρ (Figure 6E). The 0.50/200 μm fiber, however, interface with a volume approximatively two times bigger with respect to the 0.39/200 μm fiber (Figures 5D, 7B), mainly due to out-of-axis contributions. Our results suggest that the influence of the numerical aperture in defining the axial extension of the brain volume under investigation is marginal with respect to the effect of the fiber diameter. Optical fibers with smaller core, such as the 0.22/50 μm one, can be utilized to collect functional fluorescence signals from a restricted tissue volume, when localized information is needed.

From a practical perspective, our data can serve as a resource for researchers in choosing the optical fiber to use in a specific experiment: (i) increase the core size rather than the NA to enlarge the collection volume; (ii) if on-axis contribution is preferred (e.g., cortical columns), a lower NA should be used; (iii) if a low count rate is expected, high NA fibers can help by detecting additional out-of-axis fluorescence.

## Materials and Methods

### Fiber Stubs Fabrication

Fiber stubs were realized from 0.22/50 μm (Thorlabs FG050UGA), 0.39/200 μm (Thorlabs FT200UMT), and 0.50/200 μm (Thorlabs FP200URT) multimode optical with cladding diameters of 125 μm and 225 μm, for 0.22/50 μm and 0.39/200 μm−0.50/200 μm fibers, respectively. After peeling off the buffer, stubs were trimmed to size using a fiber cleaver (Fujikura CT-101) and connectorized to a stainless-steel 1.25 mm ferrule. The connectorized ends of the stubs were then manually polished. Fiber patches were realized from the same fiber types and connectorized using a stainless-steel ferrule on the proximal end and a SMA connector on the distal end, with respect to the stub. During experiments, the stubs were connected to a patch fiber of matching NA and core/cladding sizes via ferrule to ferrule butt-coupling. The patch fiber has a fully preserved core/cladding/buffer structure, that influence propagation of light collected and guided by the cladding of the fiber stub. For the 0.39/200 μm and 0.50/200 μm fibers, light entering the cladding can propagate in the patch fiber since the buffer has a refractive index *n*_buf_ < *n*_clad_. For 0.22/50 μm fiber, instead, the acrylate buffer refractive index is *n*_buf_ = 1.4950 at 520 nm (Polyanskiy, 2018), higher than *n*_clad_ = 1.4440, thus preventing light to be guided into the cladding of the patch fiber.

### Analytical Calculation of Fiber Collection Fields

Analytical 3D maps of fiber collection fields for an ideal point source were calculated for 0.22/50 μm, 0.39/200 μm, and 0.50/200 μm fibers following Engelbrecht et al. (2009). We extended their work to include the cladding front face contribution to the collection fields of 0.39/200 μm, 0.50/200 μm and 0.66/200 μm fibers by implementing Equation (1). The refractive index of the core (*n*_core_ = 1.4613 at 520 nm for 0.39 NA and 0.50 NA with silica core, and *n*_core_ = 1.5202 at 520 nm for 0.66 NA with borosilicate core) has been retrieved from the online database (Filmetrics, 2018), while the refractive index of the cladding (*n*_clad_) has been calculated to provide the nominal numerical aperture. In Equation (1), ψ (NA, *n, a, x, z*) is the same as in Ref. Engelbrecht et al. (2009), while the term ψ (NA_eq_, *n, b, x, z*) − ψ (NA_eq_, *n, a, x, z*) takes into account light collection from the cladding facet (see Supplementary Script 1). In particular, ψ (NA_eq_, *n, b, x, z*) is the collection efficiency of a fiber with diameter *b* that guides light by virtue of the refractive index contrast between the surrounding medium and the cladding with numerical aperture NA_eq_. However, the cladding has a thickness *b*-*a* with circular crown shape, and therefore the collection efficiency from the region overlapped to the core, ψ (NA_eq_, *n, a, x, z*), should be subtracted. This modeling was used for obtaining the collection efficiency maps for point-like sources shown in Figure 1B. To extend these data to the case of an extended source, three dimensional maps for point-like source were obtained by rotating collection efficiency maps in the *y* = 0 plane around the fiber axis (see Supplementary Script 1). The obtained 3D maps were then convolved with a 3D representation of the actual focal spot generated by the microscope (see Supplementary Script 1). This latter was modeled as a Gaussian function with lateral FWHM *r*_*x, z*_ = 3 μm, and axial FWHM *r*_*y*_ = 32 μm, modeling the PSF of the used experimental configuration (see below for details in the PSF measurements).

### Ray Tracing Simulation of Fiber Collection Fields

Ray tracing simulations were performed using an optical model designed with commercial optical ray-tracing software Zemax-OpticStudio to simulate the behavior of light collected by the optical fibers. The implemented layout is shown in Supplementary Figure 2. Flat fibers were represented as two nested cylinders simulating core and cladding of nominal diameters (50 μm/125 μm for 0.22NA Thorlabs FG050UGA fiber, 200 μm/225 μm for 0.39NA Thorlabs FT200UMT and 0.50NA Thorlabs FP200URT fibers, and 400 μm/425 μm). Numerical apertures of the fibers were defined by setting the respective core/cladding refractive indexes *n*_core_/*n*_clad_ as 1.4613/1.4440 for 0.22NA fibers, 1.4613/1.4079 for 0.39NA fibers, 1.4613/1.3730 for 0.50NA fibers, and 1.5202/1.3694 for 0.66NA fibers (Filmetrics, 2018). One of the two fiber facets was included within an optically homogeneous cylinder volume that simulated the PBS:fluorescein droplet or the stained brain slice. A fluorescence source was modeled as a 520 nm point source emitting 500·10^3^ rays for a total power of 1W. To reproduce the experimental acquisition, the source was raster scanned across the half-plane *y* = 0, *x* > 0 (Figure 1A). To optimize simulation time, steps along *z* and *x* were set to 25 μm; for simulations concerning 0.22/50 μm fiber the region in the proximity of the fiber (600 μm along *z* and 500 μm along *x*) was simulated with a grid step of 12.5 μm, to better sample the smaller core. For each source position, the rays were collected from both core and cladding surfaces on the facet, propagated into the fiber and they were detected on a single-pixel squared detector placed at the distal end of the fiber. The detector size was matched to the cladding diameter. Refractive indexes were set as *n* = 1.335 for PBS:fluorescein solution and as 1.360 for brain-like scattering volume (Sun et al., 2012). Scattering in the PBS:fluorescein solution was not modeled. Scattering in brain tissue was simulated following a Henyey-Greenstein model with parameters: mean free path *m* = 0.04895 mm, anisotropy value *g* = 0.9254 and transmission *T* = 0.9989 (Zinter and Levene, 2011; Yona et al., 2016).

From the computational point of view, the most demanding part of the simulation is rays propagation into the fiber, that experimentally is ~1m long and requires > 24 h per simulation. To shorten simulation times, a relatively short length of the fibers was implemented (10 mm). This short length does not allow, however, to consider losses of light entering the fiber outside the maximum accepted angle. Therefore, only rays describing an angle with the fiber input facet smaller than a threshold θ_th_ were considered in the power count, with

(4) $$\theta_{\text{th}}={\text{sin}}^{-1}\frac{\text{max}\left\{\text{NA},{\text{NA}}_{\text{eq}}\right\}}{n}.$$

For the 0.22 NA fibers the cladding sidewalls were modeled as an absorbing interface to take into account for the leakage of light from the cladding (*n*_clad_ = 1.4440 at 520 nm) to the buffer (*n*_buf_ = 1.4950 at 520 nm Polyanskiy, 2018) into the patch fiber.

### Brain Slices Treatment

Brain slices ~300 μm thick were cut with a vibratome from wild-type mice brain. Slices were then fixed in PFA and permeabilized for 30 min in 0.3% Triton X-100 (Sigma-Aldrich). Slices were then incubated with fluorescein (1mM in PBS) for 30 min.

### Acquisition and Analysis of Fiber Collection Fields

A combined confocal/two-photon laser scanning microscope was designed and built in a configuration similar to Ref. Tai et al. (2007). A full block diagram of the path used to measure collection fields is illustrated in Figure 3A. The power of a fs-pulsed near-infrared (NIR) laser beam (Coherent Chameleon Discovery, emission tuned at λ_ex_ = 920 nm) is modulated by means of a Pockels cell (Conoptics 350-80-02), and a quarter wave plate (Thorlabs AQWP05M-980) has been used to obtain circularly polarized light. The laser beam is expanded by a factor 5 and *xz*-scanned with a galvo/galvo head (Sutter). The microscope objective (Olympus XLFluor 4x/340) is mounted on a y-axis piezo focuser (Phisik Instrument P-725.4CD), and fluorescence signal is excited into a quasi-transparent 30 μM PBS:Fluorescein solution or into a fluorescently stained brain slice. Fluorescence light is re-collected by the same objective and conveyed without descanning on the entrance window of a photomultiplier tube (PMT, Hamamatsu H10770PA-40, the “μ*scope PMT*”) through a dichroic mirror (Semrock FF665-Di02), two spherical lenses (Thorlabs LA1708-A and LA1805-A), and a bandpass filter (BPF, Semrock FF01-520/70-25). During experiments in solution, fiber stubs were submerged in a PBS:Fluorescein droplet held in the sample plane by a hydrophobic layer. After a butt-to-butt coupling with a patch fiber of matched NA and core/cladding diameter, the light back emitted from the fiber was collected through a microscope objective (Olympus Plan N 40x) and sent to the entrance window of a PMT (Hamamatsu H7422P-40, the “*fiber PMT*”), through two spherical lenses (Thorlabs LA1050-A and LA1805-A) and a BPF (Semrock FF03-525/50-25).

A focal spot was then generated and scanned in the vicinity of the fiber facet covering a field of view of ~1.4 × 1.4 mm^2^ with 512 × 512 pixels, with the beam resting on each pixel for ~3.2 μs. Laser power and PMTs gain were adjusted to optimize signal to noise ratio. For each measurement, a 400 μm thick stack was acquired with a 5 μm step along *y*, starting slightly below the fiber axis and finishing above the fiber. Each slice in the stack was averaged out of 5 frames.

The number of photons for each frame was calculated as $N_{ph}=\frac{PMT_{\text{counts}}}{G}$, where *G* represents the gain of the acquisition system. *G* was measured as $G=\sigma_{\text{counts}}^{2}/\langle PMT_{\text{counts}}\rangle$, where the average number of counts 〈*PMT*_counts_〉 and the variance $\sigma_{\text{counts}}^{2}$ were acquired by illuminating a confined and homogeneous region in the fluorescein drop. Stacks acquired through the *fiber PMT* were corrected slice-by-slice for unevenness in excitation, by scaling them against the normalized corresponding image collected by the μ*scope PMT* (see Supplementary Figure 5). The frame acquired for gain measurement of the epi-fluorescence path has been used to correct for slight variability of laser power between measurements, proportionally to the pixel average value. Uncertainty σ_c_ on the cumulative number of photons shown in Figure 4C were evaluated propagating the Poisson

(5) $$\sigma_{c}\left(z\right)=\sqrt{\sum_{x,y,z}{\left\{N_{ph}\left(x,y,z\right)\sqrt{{\left(\frac{1}{\sqrt{PMT_{\text{counts}}^{\text{fiber}}\left(x,y,z\right)}}\right)}^{2}+{\left(\frac{1}{\sqrt{PMT_{\text{counts}}^{\mu \text{​scope}}\left(x,y,z\right)}}\right)}^{2}+{\left[\frac{\text{std}\left(G^{\text{fiber}}\right)}{\langle G^{\text{fiber}}\rangle }\right]}^{2}}\right\}}^{2}}$$

noise on the photon count of every pixel and the error on gain determination as where the superscript *fiber* and μ*scope* identify the PMT, mean and standard deviation of *G* are evaluated over five consecutive frames, and the sum indexes span across the whole *xy* plane and up to *z*. The value of σ_c_ resulted to be <1% of *N*_c_ at all *z* for all fibers. Collection efficiency η was evaluated pixel by pixel as

(6) $$\begin{array}{l} \;\eta \left(x,y,z\right)\\ =\frac{PMT_{\text{counts}}^{\text{fiber}}\left(x,y,z\right)/G^{\text{fiber}}}{PMT_{\text{counts}}^{\mu \text{​scope}}\left(x,y,z\right)/\left[G^{\mu \text{​scope}}\cdot 0.5\gamma \left(1-\text{cos}\left(NA_{\text{obj}}/n\right)\right)\right]}, \end{array}$$

where γ = 1.11 is a factor compensating for the loss in the patch fiber and the term 0.5(1 − cos(*NA*_obj_/*n*)) is the fraction of solid angle accepted by the microscope objective. Data processing was done through Supplementary Script 2 and Supplementary Script 3 for images collected in quasi-transparent medium and in brain slice, respectively. One 0.22NA/50 μm fiber, four 0.39NA/200 μm and four 0.50NA/200 μm fibers were characterized in quasi-transparent medium, three 0.39NA/200 μm and three 0.50NA/200 μm fibers were characterized in brain slice; data among the same nominal fiber were averaged, and twice the standard deviation was considered as error bar.

### Point Spread Function Measurement

The PSF of the two-photon microscope was measured by imaging sub-resolution nanoparticles (100 nm) at 920 nm with 160 nm lateral steps and 2 μm axial steps. For the 4X/0.28NA Olympus XLFluor 4x/340 objective, this resulted in a PSF with lateral FWHM *r*_*x, z*_ = 3 μm ± 1 μm, and axial FWHM *r*_*y*_ = 32 μm ± 5 μm (Supplementary Figure 3). Lateral and axial profiles were fitted with a gaussian function. Two nanoparticles were considered in this measurement, values shown are mean ± standard deviation.

### Acquisition of Spatially Sampled Fiber Emission Diagrams

The setup schematically shown in Figure 6A was used to measure the emission diagrams of flat-cleaved optical fibers in tissue. Fibers were inserted into a 300 μm thick fluorescently stained brain slice, 473 nm light was coupled into the fiber through an objective lens (Olympus Plan N 40x), and the primary dichroic of the 2P microscope was removed from the system. Light emission from the tissue was collected through the microscope objective, descanned by the scan-head, focused into a pinhole (Thorlabs MPH-16), and detected by a PMT (Hamamatsu H7422P-40, the “*pinhole PMT*”). A BPF (Thorlabs MF525/39) isolated the wavelength band of interest. The pinhole size was set to 100 μm.

### Photometry Efficiency Calculation

Images acquired on the *y* = 0 plane by the *fiber PMT* and the *pinhole PMT* were used to determine the photometry efficiency. The image acquired through the pinhole was registered over the collection field to obtain a pixel-to-pixel spatial correspondence. The photometry efficiency maps were determined as the pixel by pixel product of normalized version of illumination and collection fields (see Supplementary Script 4). One half of the photometry efficiency maps was employed to obtain a volumetric representation of this quantity, as reported previously.

### Matlab Programming

Data processing was implemented in Matlab. Scripts are reported in Supplementary Materials, with Supplementary Script 5 containing all the undefined functions called in Supplementary Scripts 1–4.

## Data Availability

Data will be made available by authors after the publication of the paper at cbn.iit.it/openfiberphotometry and can be requested to the authors at any time.

## Author Contributions

All authors listed have made a substantial, direct and intellectual contribution to the work, and approved it for publication.

### Conflict of Interest Statement

The authors declare that the research was conducted in the absence of any commercial or financial relationships that could be construed as a potential conflict of interest.

## Supplementary Material

Supplementary Video 1 Volumetric scan of the collection field of a 0.50NA/200 μm fiber in a 30 μm PBS: Fluorescein solution.

## References

[B1] Adelsberger H. Zainos A. Alvarez M. Romo R. Konnerth A. (2014). Local domains of motor cortical activity revealed by fiber-optic calcium recordings in behaving nonhuman primates. *Proc. Natl. Acad*. Sci.U.S.A. 111, 463–468. 10.1073/pnas.1321612111 PMC3890885 24344287

[B2] Aravanis A. M. Wang L. P. Zhang F. Meltzer L. A. Mogri M. Z. Schneider M. B. (2007). An optical neural interface: *in vivo* control of rodent motor cortex with integrated fiberoptic and optogenetic technology. *J*. Neural Eng. 4, S143–S156. 10.1088/1741-2560/4/3/S02 17873414

[B3] Bargo P. R. Prahl S. A. Jacques S. L. (2002). Collection efficiency of a single optical fiber in turbid media for reflectance spectroscopy, in Biomedical Topical Meeting (Optical Society of America), 604–606.

[B4] Bargo P. R. Prahl S. A. Jacques S. L. (2003a). Collection efficiency of a single optical fiber in turbid media. *Appl*. Opt. 42, 3187–3197. 10.1364/AO.42.003187 12790469

[B5] Bargo P. R. Prahl S. A. Jacques S. L. (2003b). Optical properties effects upon the collection efficiency of optical fibers in different probe configurations. *IEEE J. Sel. Top. Q*. Electron. 9, 314–321. 10.1109/JSTQE.2003.811287

[B6] Canales A. Jia X. Froriep U. P. Koppes R. A. Tringides C. M. Selvidge J. (2015). Multifunctional fibers for simultaneous optical, electrical and chemical interrogation of neural circuits *in vivo. Nat*. Biotechnol. 33, 277–284. 10.1038/nbt.3093 25599177

[B7] Chen Y. Lin Y. C. Kuo T. W. Knight Z. A. (2015). Sensory detection of food rapidly modulates arcuate feeding circuits. Cell 160, 829–841. 10.1016/j.cell.2015.01.033 25703096 PMC4373539

[B8] Cui G. Jun S. B. Jin X. Luo G. Pham M. D. Lovinger D. M. (2014). Deep brain optical measurements of cell type–specific neural activity in behaving mice. *Nat*. Protoc. 9, 1213–1228. 10.1038/nprot.2014.080 PMC4100551 24784819

[B9] Cui G. Jun S. B. Jin X. Pham M. D. Vogel S. S. Lovinger D. M.. (2013). Concurrent activation of striatal direct and indirect pathways during action initiation. Nature 494, 238–242. 10.1038/nature11846 23354054 PMC4039389

[B10] Deisseroth K. (2011). Optogenetics. *Nat*. Methods 8, 26–29. 10.1038/nmeth.f.324 PMC6814250 21191368

[B11] Emiliani V. Cohen A. E. Deisseroth K. Hausser M. (2015). All-optical interrogation of neural circuits. *J*. Neurosci. 35, 13917–13926. 10.1523/JNEUROSCI.2916-15.2015 PMC4604230 26468193

[B12] Engelbrecht C. J. Göbel W. Helmchen F. (2009). Enhanced fluorescence signal in nonlinear microscopy through supplementary fiber-optic light collection. *Opt*. Express. 17, 6421–6435. 10.1364/OE.17.006421 19365467

[B13] Filmetrics (2018). Refractive Index Database. Available online at: https://www.filmetrics.com/refractive-index-database

[B14] Fluhler E. Burnham V. G. Loew L. M. (1985). Spectra, membrane binding, and potentiometric responses of new charge shift probes Biochemistry 24, 5749–5755. 10.1021/bi00342a010 4084490

[B15] Fuhrmann F. Justus D. Sosulina L. Kaneko H. Beutel T. Friedrichs D.. (2015). Locomotion, theta oscillations, and the speed-correlated firing of hippocampal neurons are controlled by a medial septal glutamatergic circuit. Neuron 86, 1253–1264. 10.1016/j.neuron.2015.05.001 25982367

[B16] Goßler C. Bierbrauer C. Moser R. Kunzer M. Holc K. Pletschen W. (2014). GaN-based micro-LED arrays on flexible substrates for optical cochlear implants. J. Phys. D. Appl. Phys. 47:205401 10.1088/0022-3727/47/20/205401

[B17] Grienberger C. Adelsberger H. Stroh A. Milos R. I. Garaschuk O. Schierloh A. (2012). Sound-evoked network calcium transients in mouse auditory cortex *in vivo. J*. Physiol. 590, 899–918. 10.1113/jphysiol.2011.222513 PMC3381318 22106174

[B18] Gunaydin L. A. Grosenick L. Finkelstein J. C. Kauvar I. V. Fenno L. E. Adhikari A.. (2014). Natural neural projection dynamics underlying social behavior. Cell 157, 1535–1551. 10.1016/j.cell.2014.05.017 24949967 PMC4123133

[B19] Häusser M. (2014). Optogenetics: the age of light. *Nat*. Methods. 11, 1012–1014. 10.1038/nmeth.3111 25264778

[B20] He Y. Wang M. Chen X. Pohmann R. Polimeni J. R. Scheffler K.. (2018). Ultra-slow single-vessel BOLD and CBV-based fMRI spatiotemporal dynamics and their correlation with neuronal intracellular calcium signals. Neuron 97, 925–939.e5. 10.1016/j.neuron.2018.01.025 29398359 PMC5845844

[B21] Kim C. K. Yang S. J. Pichamoorthy N. Young N. P. Kauvar I. Jennings J. H. (2016). Simultaneous fast measurement of circuit dynamics at multiple sites across the mammalian brain. *Nat*. Methods 13, 325–328. 10.1038/nmeth.3770 PMC5717315 26878381

[B22] Kim T. I. McCall J. G. Jung Y. H. Huang X. Siuda E. R. Li Y.. (2013). Injectable, cellular-scale optoelectronics with applications for wireless optogenetics. Science 340, 211–216. 10.1126/science.1232437 23580530 PMC3769938

[B23] Loew L. M. (1996). Potentiometric dyes: Imaging electrical activity of cell membranes. *Pure Appl*. Chem. 68, 1405–1409. 10.1351/pac199668071405

[B24] Lovett-Barron M. Andalman A. S. Allen W. E. Vesuna S. Kauvar I. Burns V. M.. Ancestral circuits for the coordinated modulation of brain state. Cell (2017). 171:1411–1423.e17. 10.1016/j.cell.2017.10.021 29103613 PMC5725395

[B25] Lu L. Gutruf P. Xia L. Bhatti D. L. Wang X. Vazquez-Guardado A. (2018). Wireless optoelectronic photometers for monitoring neuronal dynamics in the deep brain. *Proc. Natl. Acad. Sci*. U.S.A. 115, E1374–E1383. 10.1073/pnas.1718721115 PMC5816195 29378934

[B26] Luo L. Callaway E. M. Svoboda K. (2018). Genetic dissection of neural circuits: a decade of progress. Neuron 98, 256–281. 10.1016/j.neuron.2018.03.040 29673479 PMC5912347

[B27] Lütcke H. Murayama M. Hahn T. Margolis D. J. Astori S. Zum Alten Borgloh S. M. (2010). Optical recording of neuronal activity with a genetically-encoded calcium indicator in anesthetized and freely moving mice. *Front*. Neural Circuits 4:9 10.3389/fncir.2010.00009 PMC2866455 20461230

[B28] Matthews G. A. Nieh E. H. Vander Weele C. M. Halbert S. A. Pradhan R. V. Yosafat A. S.. (2016). Dorsal raphe dopamine neurons represent the experience of social isolation. Cell 164, 617–631. 10.1016/j.cell.2015.12.040 26871628 PMC4752823

[B29] McAlinden N. Gu E. Dawson M. D. Sakata S. Mathieson K. (2015). Optogenetic activation of neocortical neurons *in vivo* with a sapphire-based micro-scale LED probe, Front. Neural Circuits 9:25 10.3389/fncir.2015.00025 PMC4448043 26074778

[B30] McAlinden N. Massoubre D. Richardson E. Gu E. Sakata S. Dawson M. D. (2013). Thermal and optical characterization of micro-LED probes for *in vivo* optogenetic neural stimulation. *Opt*. Lett. 38, 992 10.1364/OL.38.000992 23503284

[B31] Meng C. Zhou J. Papaneri A. Peddada T. Xu K. Cui G. (2018). Spectrally resolved fiber photometry for multi-component analysis of brain circuits. Neuron 98, 707–717.e4. 10.1016/j.neuron.2018.04.012 29731250 PMC5957785

[B32] Miesenböck G. (2009). The optogenetic catechism. Science 326, 395–399. 10.1126/science.1174520 19833960

[B33] Miyawaki A. Llopis J. Heim R. McCaffery J. M. Adams J. A. Ikura M.. (1997). Fluorescent indicators for Ca2+based on green fluorescent proteins and calmodulin. Nature 388, 882–887. 10.1038/42264 9278050

[B34] Muir J. Lorsch Z. S. Ramakrishnan C. Deisseroth K. Nestler E. J. Calipari E. S.. (2017). *In vivo* fiber photometry reveals signature of future stress susceptibility in nucleus accumbens. Neuropsychopharmacology 43, 255–263. 10.1038/npp.2017.122 28589967 PMC5729554

[B35] Nieh E. H. Vander Weele C. M. Matthews G. A. Presbrey K. N. Wichmann R. Leppla C. A.. (2016). Inhibitory input from the lateral hypothalamus to the ventral tegmental area disinhibits dopamine neurons and promotes behavioral activation. Neuron 90, 1286–1298. 10.1016/j.neuron.2016.04.035 27238864 PMC4961212

[B36] Park S. Guo Y. Jia X. Kyoung Choe H. Grena B. Kang J. (2017). One-step optogenetics with multifunctional flexible polymer fibers. *Nat*. Neurosci. 20, 612–619. 10.1038/nn.4510 PMC5374019 28218915

[B37] Petersen C. C. Grinvald A. Sakmann B. (2003). Spatiotemporal dynamics of sensory responses in Layer 2/3 of rat barrel cortex measured *in vivo* by voltage-sensitive dye imaging combined with whole-cell voltage recordings and neuron reconstructions. *J*. Neurosci. 23, 1298–1309. 10.1523/JNEUROSCI.23-04-01298.2003 PMC6742278 12598618

[B38] Pfefer T. Schomacker K. Ediger M. Nishioka N. S. (2001). Light propagation in tissue during fluorescence spectroscopy withsingle-fiber probes. *IEEE J. Sel. Top. Q*. Electron. 7, 1004–1012. 10.1109/2944.983306

[B39] Pfefer T. J. Schomacker K. T. Ediger M. N. Nishioka N. S. (2002). Multiple-fiber probe design for fluorescence spectroscopy in tissue. *Appl*. Opt. 41, 4712–4721. 10.1364/AO.41.004712 12153108

[B40] Pisanello F. Mandelbaum G. Pisanello M. Oldenburg I. A. Sileo L. Markowitz J. E.. (2017). Dynamic illumination of spatially restricted or large brain volumes via a single tapered optical fiber. *Nat*. Neurosci. 20, 1180–1188. 10.1038/nn.4591 28628101 PMC5533215

[B41] Pisanello F. Sileo L. Oldenburg I. A. Pisanello M. Martiradonna L. Assad J. A.. (2014). Multipoint-emitting optical fibers for spatially addressable *in vivo* optogenetics. Neuron 82, 1245–1254. 10.1016/j.neuron.2014.04.041 24881834 PMC4256382

[B42] Pisanello M. Pisano F. Sileo L. Maglie E. Bellistri E. Spagnolo B.. (2018). Tailoring light delivery for optogenetics by modal demultiplexing in tapered optical fibers. Sci. Rep. 8:4467. 10.1038/s41598-018-22790-z 29535413 PMC5849750

[B43] Pisano F. Pisanello M. Sileo L. Qualtieri A. Sabatini B. L. De Vittorio M. (2018). Focused ion beam nanomachining of tapered optical fibers for patterned light delivery. *Microelectron*. Eng. 195, 41–49. 10.1016/j.mee.2018.03.023 PMC6565430 31198228

[B44] Polyanskiy M. N. (2018). Refractive Index Database. Available Online at: https://refractiveindex.info

[B45] Ryu Y. Shin Y. Lee D. Altarejos J. Y. Chung E. Kwon H. S. (2015). Lensed fiber-optic probe design for efficient photon collection in scattering media. *Biomed. Opt*. Express 6:191 10.1364/BOE.6.000191 PMC4317131 25657886

[B46] Scharf R. Tsunematsu T. McAlinden N. Dawson M. D. Sakata S. Mathieson K. (2016). Depth-specific optogenetic control *in vivo* with a scalable, high-density μlED neural probe. *Sci*. Rep. 6:28381 10.1038/srep28381 PMC4917834 27334849

[B47] Schmid F. Wachsmuth L. Schwalm M. Prouvot P. H. Jubal E. R. Fois C. (2016). Assessing sensory versus optogenetic network activation by combining (o)fMRI with optical Ca 2+ recordings. *J. Cereb*. Blood Flow Metab. 36, 1885–1900. 10.1177/0271678X15619428 PMC5094300 26661247

[B48] Schwalm M. Schmid F. Wachsmuth L. Backhaus H. Kronfeld A. Aedo Jury F.. (2017). Cortex-wide BOLD fMRI activity reflects locally-recorded slow oscillation-associated calcium waves. Elife 6:e27602. 10.7554/eLife.27602 28914607 PMC5658067

[B49] Segev E. Reimer J. Moreaux L. C. Fowler T. M. Chi D. Sacher W. D.. (2016). Patterned photostimulation via visible-wavelength photonic probes for deep brain optogenetics. Neurophotonics 4:011002. 10.1117/1.NPh.4.1.011002 27990451 PMC5136672

[B50] Selimbeyoglu A. Kim C. K. Inoue M. Lee S. Y. Hong A. S. O. Kauvar I.. (2017). Modulation of prefrontal cortex excitation/inhibition balance rescues social behavior in CNTNAP2 -deficient mice. Sci. Transl. Med. 9:eaah6733. 10.1126/scitranslmed.aah6733 28768803 PMC5723386

[B51] Simone K. Füzesi T. Rosenegger D. Bains J. Murari K., (2018). Open-source, cost-effective system for low-light *in vivo* fiber photometry. Neurophotonics 5:025006. 10.1117/1.NPh.5.2.025006 29687037 PMC5895965

[B52] Slovin H. Arieli A. Hildesheim R. Grinvald A. (2002). Long-term voltage-sensitive dye imaging reveals cortical dynamics in behaving monkeys. *J*. Neurophysiol. 88, 3421–3438. 10.1152/jn.00194.2002 12466458

[B53] Snyder A. W. Love J. D. (1983). Optical Waveguide Theory. New York, NY: Chapman and Hall.

[B54] Stroh A. Adelsberger H. Groh A. Rühlmann C. Fischer S. Schierloh A.. (2013). Making waves: initiation and propagation of corticothalamic Ca2+ waves in vivo Neuron 77, 1136–1150. 10.1016/j.neuron.2013.01.031 23522048

[B55] Sun J. Lee S. J. Wu L. Sarntinoranont M. Xie H. (2012). Refractive index measurement of acute rat brain tissue slices using optical coherence tomography. *Opt*. Express. 20:1084 10.1364/OE.20.001084 PMC3501791 22274454

[B56] Tai D. C. Hooks D. A. Harvey J. D. Smaill B. H. Soeller C. (2007). Illumination and fluorescence collection volumes for fiber optic probes in tissue. *J. Biomed*. Opt. 12:034033 10.1117/1.2750288 17614741

[B57] Wu F. Stark E. Ku P. C. Wise K. D. Buzsáki G. Yoon E. (2015). Monolithically integrated μLEDs on silicon neural probes for high-resolution optogenetic studies in behaving animals. Neuron 88, 1136–1148. 10.1016/j.neuron.2015.10.032 26627311 PMC4702503

[B58] Yizhar O. Fenno L. E. Davidson T. J. Mogri M. Z. Deisseroth K. (2011). Optogenetics in neural systems. Neuron 71, 9–34. 10.1016/j.neuron.2011.06.004 21745635

[B59] Yona G. Weissler Y. Meitav N. Guzi E. Rifold D. Kahn I. (2016). Realistic modeling of optogenetic neuronal excitation in light-scattering brain tissue. Biomed. Opt. 2016:JW3A21. 10.1364/CANCER.2016.JW3A.21

[B60] Zhu Z. Y. Yappert M. C. (1992). Determination of effective depth and equivalent pathlength for a single-fiber fluorometric sensor. *Appl*. Spectrosc. 46, 912–918. 10.1366/0003702924124411

[B61] Zinter J. P. Levene M. J. (2011). Maximizing fluorescence collection efficiency in multiphoton microscopy. *Opt*. Express 19, 15348 10.1364/OE.19.015348 PMC3482884 21934897

[B62] Zorzos A. N. Scholvin J. Boyden E. S. Fonstad C. G. (2012). Three-dimensional multiwaveguide probe array for light delivery to distributed brain circuits. *Opt*. Lett. 37:4841 10.1364/OL.37.004841 PMC3572236 23202064

