# Supplementary material for: The Three-Dimensional Signal Collection Field for Fiber Photometry in Brain Tissue
Source: Front Neurosci. 2019 Feb 26;13:82. doi: 10.3389/fnins.2019.00082 (PMC6399578; doi:10.3389/fnins.2019.00082)
Supplement: Supplementary file 2 [file Data_Sheet_1.docx]

Supplementary Material

The three-dimensional signal collection field for fiber photometry in brain tissue

Marco Pisanello^1+*^, Filippo Pisano^1+^, Minsuk Hyun^2+^, Emanuela Maglie^1,3^, Antonio Balena^1,3^, Massimo De Vittorio^1,3^, Bernardo L. Sabatini^2*^, Ferruccio Pisanello^1*^

^1^Istituto Italiano di Tecnologia, Center for Biomolecular Nanotechnologies, Arnesano, Lecce, Italy

^2^Department of Neurobiology, Howard Hughes Medical Institute, Harvard Medical School, Boston, MA, U.S.A.

^+^Equally contributed to the work

*** Correspondence:**Corresponding Authors
marco.pisanello@iit.it
bernardo_sabatini@hms.harvard.edu
ferruccio.pisanello@iit.it

# Supplementary Figures


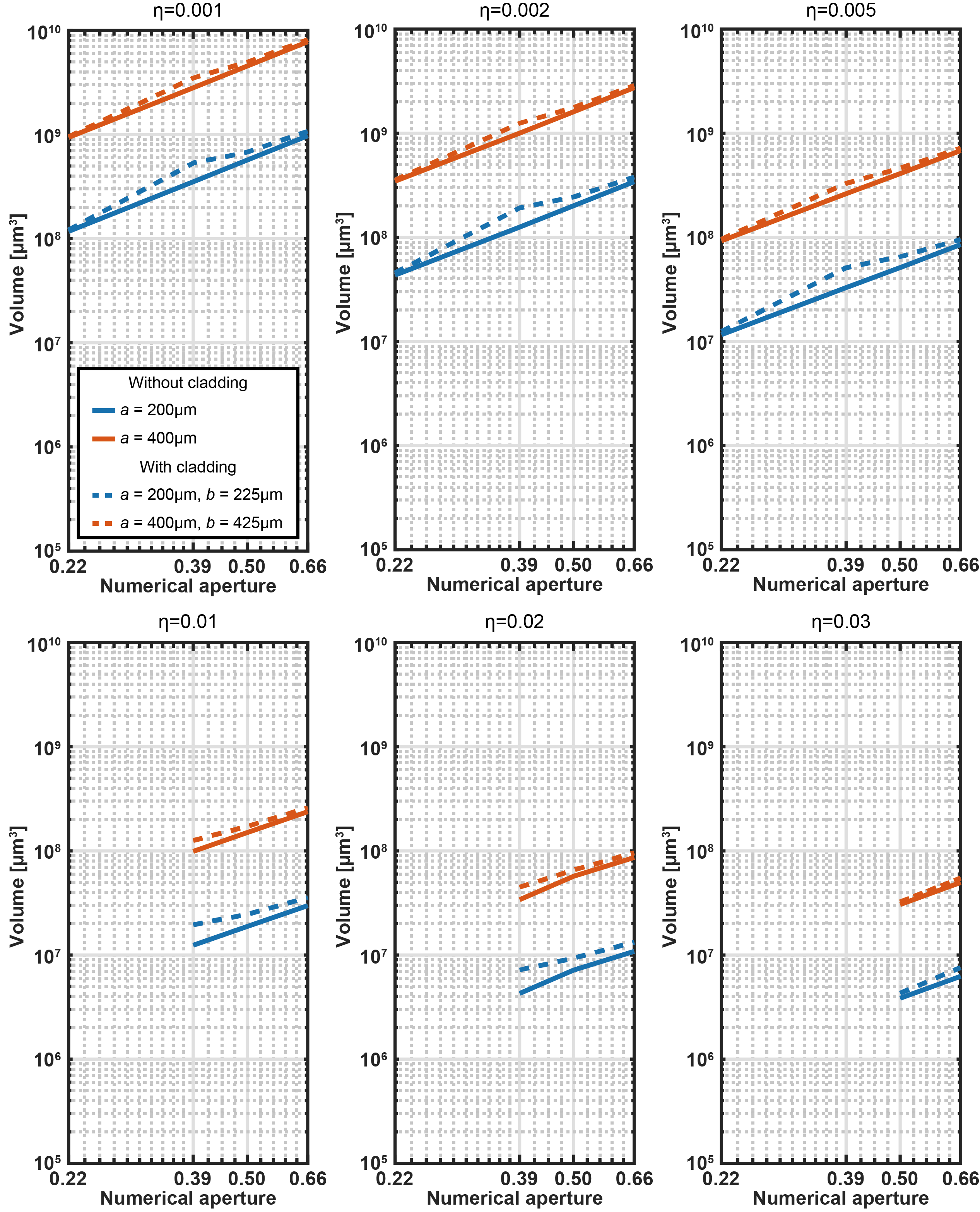


**Supplementary Figure 1.** Comparison between the volumes enclosed by iso-surfaces at *η*= 0.001, 0.002, 0.005, 0.01, 0.02, 0.03 for fibers with NA = 0.22, 0.39, 0.50, 0.66 and core/cladding diameters *a*/*b* = 200µm/225µm, 400µm/425µm (blue and red curves, respectively) with and without considering the effect of the cladding (dashed and continuous lines, respectively). Missing data at low NA for *η*= 0.01, 0.02, 0.03 correspond to null volumes.


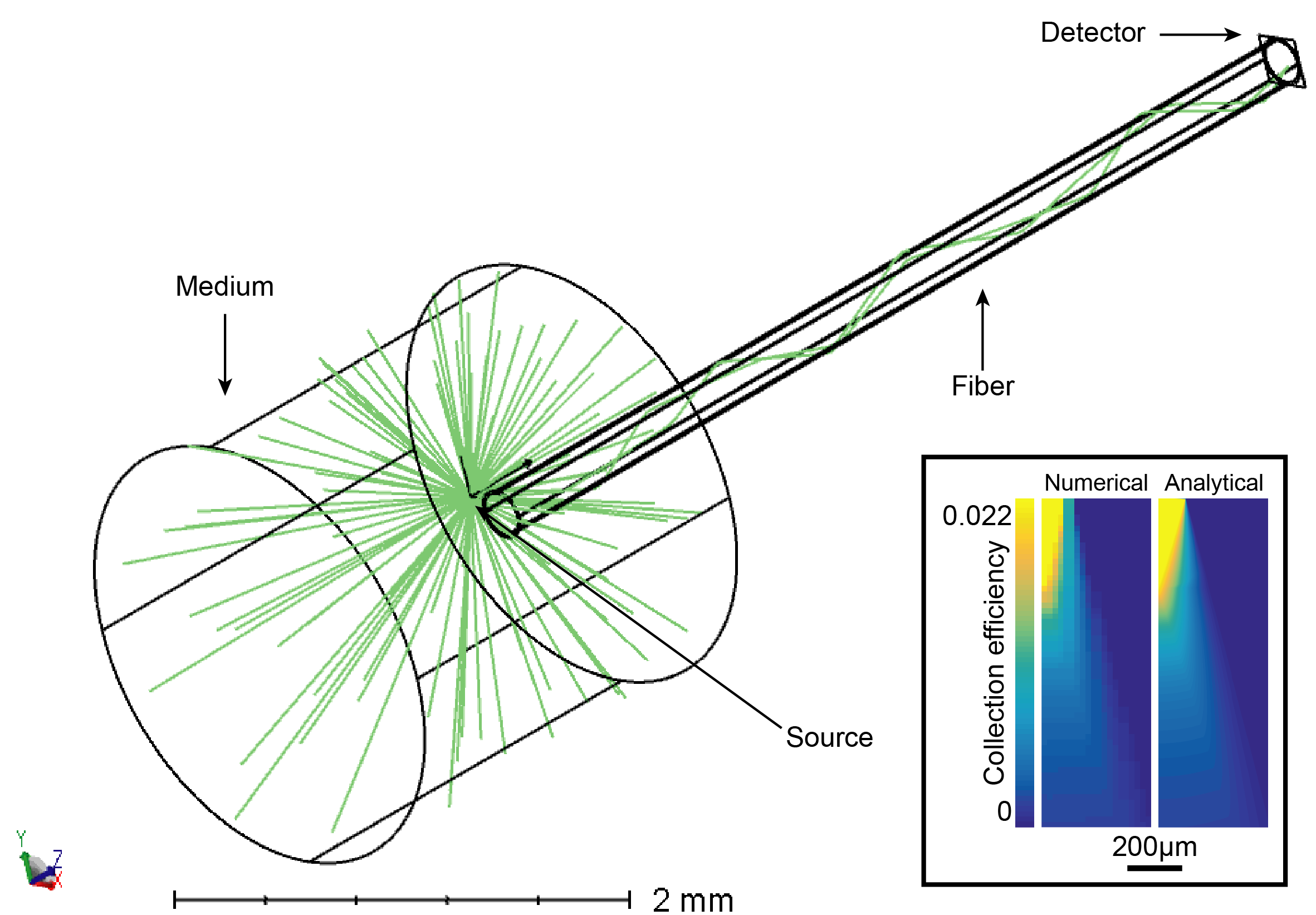


**Supplementary Figure 2.** Ray tracing layout designed to numerically evaluate light collection efficiency of optical fibers. The inset shows a comparison of collection field obtained through ray-tracing simulation and analytical model neglecting the cladding effect [Engelbrecht, et al, 2009] for a 0.39NA/200µm fiber.


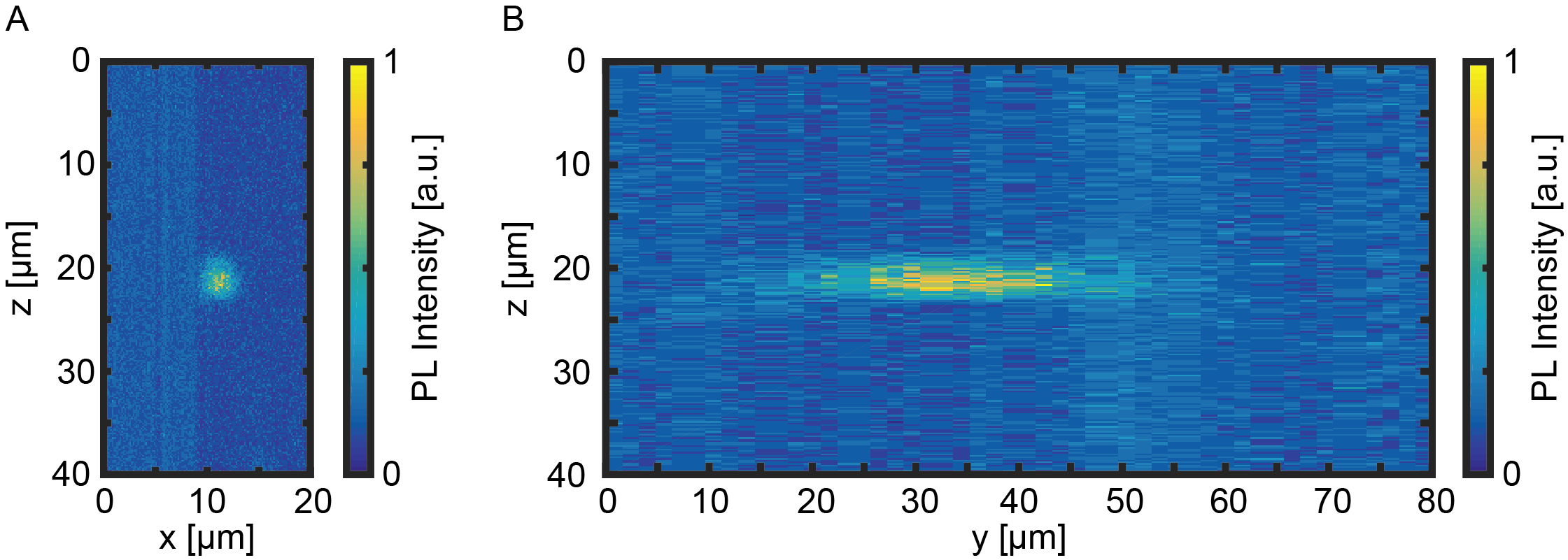


**Supplementary Figure 3.** A, Lateral section of the custom two-photon microscope point spread function. B, Axial section of the custom two-photon microscope point spread function.


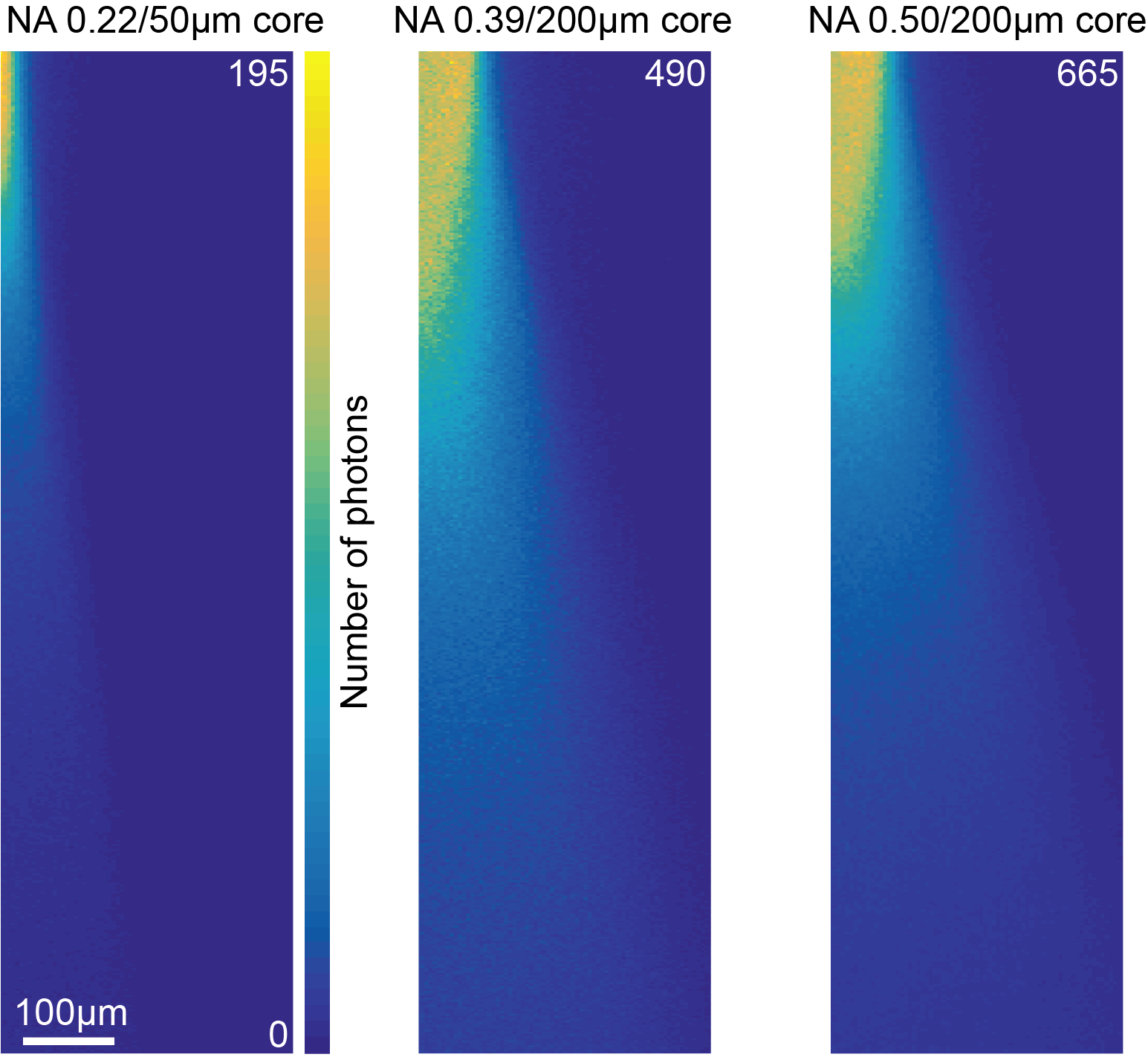


**Supplementary Figure 4.** Section *x*= 0 of the collection diagram of 0.22/50µm, 0.39/200µm, and 0.50/200µm optical fibers, as indicated, in a 30µM PBS:fluorescein solution, obtained through the *fiber PMT* as shown in Fig. 3A. The number in the top-right corner in each panel represents the maximum Number of photons relative to the color scale.


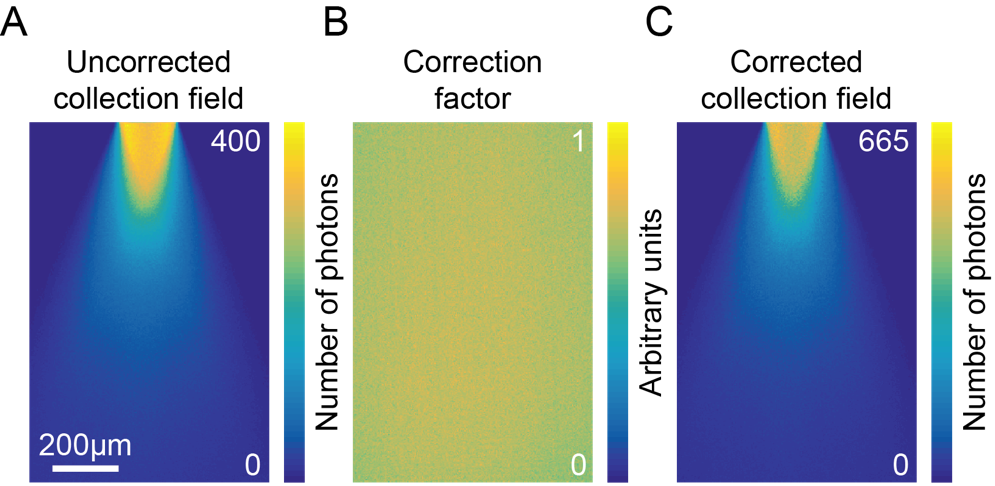


**Supplementary Figure 5.** Example of correction for unevenness of illumination within the field of view for a 0.50NA/200µm optical fiber. A, uncorrected signal collected by the *fiber PMT*. B, normalized signal collected by the *µscope PMT*. C, corrected signal collected by the *fiber PMT* as ratio between the maps in panels A and B.


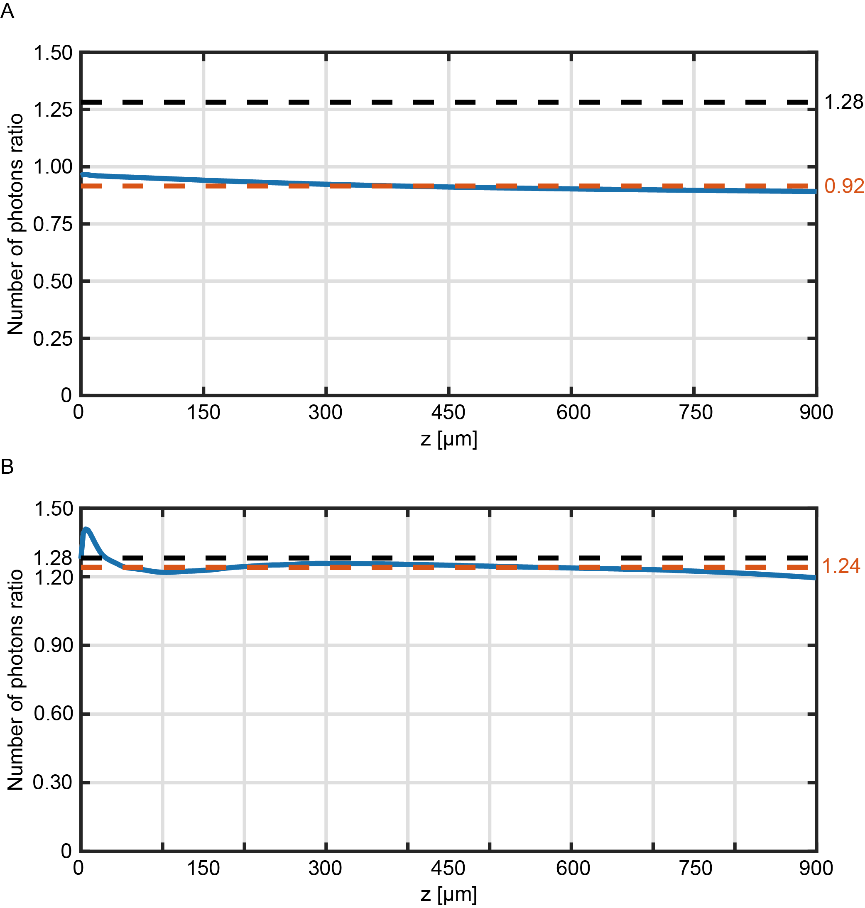


**Supplementary Figure 6.** A, Ratio of the number of collected photons within a certain depth between 0.50NA/200µm and 0.39/200µm fibers in quasi-transparent medium (blue curve); the dashed orange line represents the average signal over the entire 900µm depth, the black dashed line represents the ratio between NAs (0.50/0.39~1.28). B, Ratio of the numbers of collected photons within a certain depth between 0.50NA/200µm and 0.39/200µm fibers in brain slice (blue curve); the dashed orange line represents the average signal over the entire 900µm depth, the black dashed lines represent the ratio between NAs.


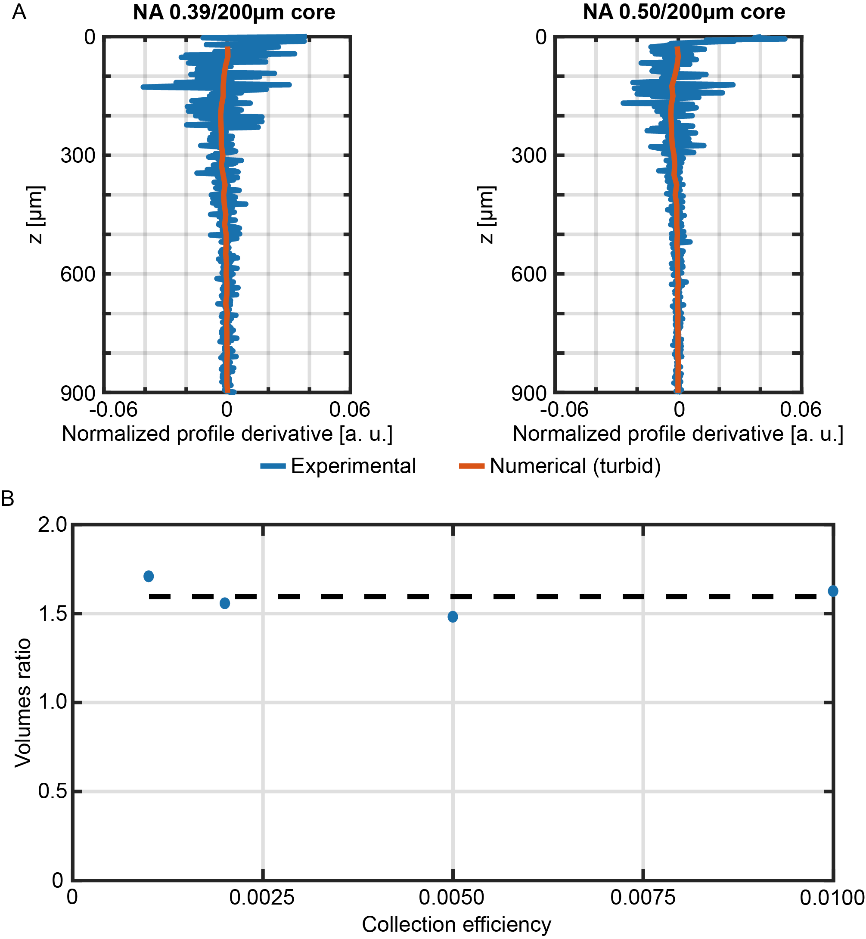


**Supplementary Figure 7.** A, Comparison of the normalized axial profiles derivatives (*x* = 0, *y* = 0) between experimental and numerical data for 0.39/200µm and 0.50/200µm optical fibers (left and right panels, respectively) in turbid media. B, Ratio of the volumes enclosed by the iso-intensity surfaces at fixed *η* (0.50/200µm fiber over 0.39/200µm fiber); the dashed line represents the average of the data points.


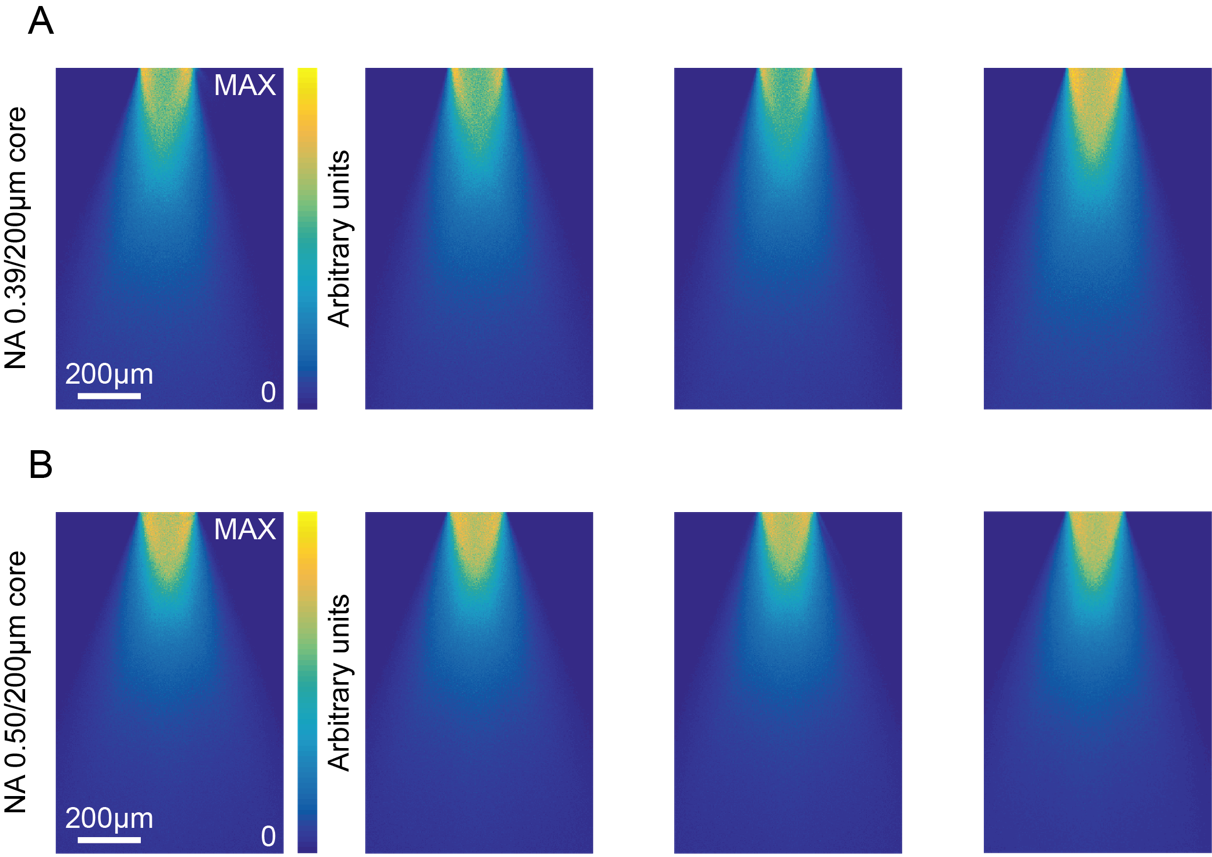


**Supplementary Figure 8.** Section *y* = 0 of the collection field of four different 0.39/200µm and 0.50/200µm optical fibers (panel A and B, respectively) in a 30µM PBS:fluorescein solution, obtained through the *fiber PMT*.


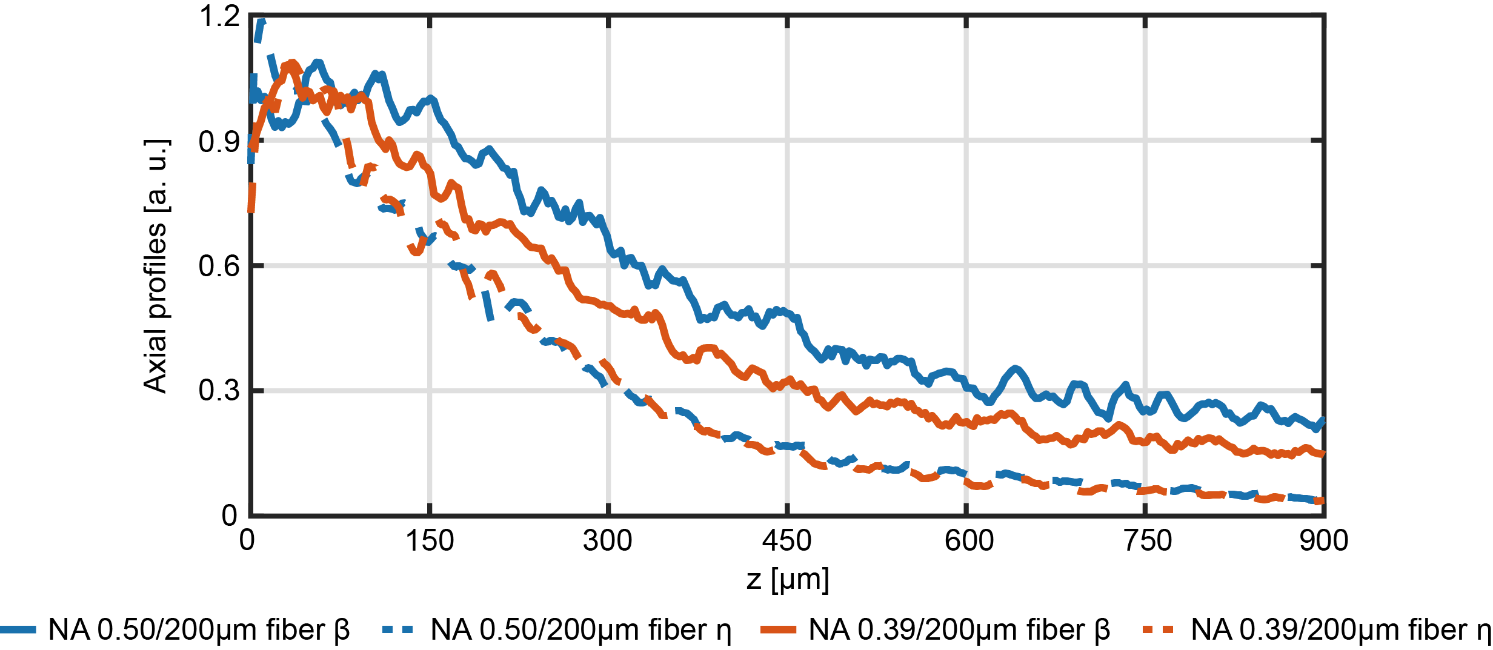


**Supplementary Figure 9.** Difference between emission and collection fields axial profiles (continuous and dashed curve, respectively) for 0.39NA/200µm and 0.50NA/200µm fibers (orange and red curve, respectively).


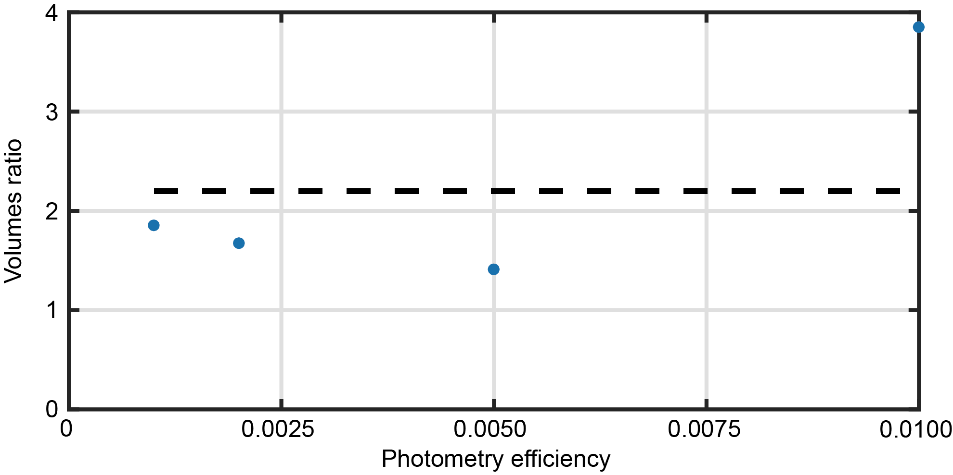


**Supplementary Figure 10.** Ratio of the volumes enclosed by the iso-intensity surfaces at fixed *ρ* (0.50/200µm fiber over 0.39/200µm fiber); the dashed line represents the average of the data points.

# Supplementary Script

**Supplementary Script 1: analytical evaluation of collection fields**

function eta=maps_eta_halfplane(NA,a,n,r,z,visualize)

%maps_eta_halfplane(NA,a,n,r,z) calculate the fiber collection efficiency according

%to Engelbrecht et al. 2009 (doi: 10.1364/OE.17.006421).

%INPUT PARAMETERS

%NA: fiber numerical aperture.

%a: fiber core diameter (in micrometers).

%n: medium refractive index.

%r: radial extension of calculation domain (in micrometers).

%z: axial extension of calculation domain (in micrometers).

%visualize: if equal to 1, the map is plotted.

%OUTPUT VARIABLES

%eta: 2D map of collection efficiency.

[rg,zg]=meshgrid(r,z);

a=a/2;

%Calculates the acceptance cone angle

alfa=asin(NA/n);

%Calculates z0

z0=a/tan(alfa);

%Calculates the solid angle of the acceptance cone

omegaNA=2*pi*(1-cos(alfa));

%Calculates the solid angle of the cone with vertex in an arbitrary

%position and base on the core facet

omegaf=2*pi*(1-cos(atan(a./zg.*cos(atan(rg./zg)).^(3/2))));

%Calculates the solid angle in which emitted power can be accepted by

%the fiber

omega=min(omegaf,omegaNA*ones(size(zg)));

%Normalized coordinates

u=r/a;

v=z/z0;

[ug,vg]=meshgrid(u,v);

%Calculates the fraction of useful optical fiber reaching the core

%facet

P1=acos((ug.^2-vg.^2+1)./(2*ug));

P2=vg.^2.*acos((ug.^2+vg.^2-1)./(2*vg.*ug));

P3=-1/2*sqrt((1-(vg-ug).^2).*((vg+ug).^2-1));

af=real(P1+P2+P3)./(pi*min(vg,ones(size(vg))).^2);

af(ug>vg+1)=0;

af(ug<=max(vg,ones(size(vg)))-min(vg,ones(size(vg))))=1;

%Calculates collection efficiency

eta=1/(4*pi)*omega.*af;

%Visualization of the results.

if visualize==1

figure(1);

imagesc(r,z,eta);

set(gca,'FontSize',20,'FontWeight','bold','LineWidth',2.5);

colorbar('LineWidth',2.5);

xlabel('r [\mum]','FontWeight','bold');

ylabel('z [\mum]','FontWeight','bold');

title(strcat('Collection efficiency of',{' '},num2str(NA),'NA/',num2str(2*a),'\mum core fibers in n=',num2str(n),' medium'));

axis equal;

axis([min(r) max(r) min(z) max(z)]);

display(strcat('Collection efficiency of',{' '},num2str(NA),'NA/',num2str(2*a),'\mum core fibers in n=',num2str(n),' medium'));

display(strcat('Maximum efficiency:',{' '},num2str(max(max(eta)))));

display(strcat('NA regime distance:',{' '},num2str(z0),'um'));

end

end

function [eta_pointsource,eta_convolved]=volume_eta(NA,core_diameter,cladding_diameter,ncore,n,r_eta,z_eta,source)

%volume_eta evaluates the analytical 3D collection efficiency diagram for

%both point-like and extended source in quasi transparent medium.

%INPUT PARAMETERS

%NA: fiber numerical aperture.

%core_diameter: fiber core diameter.

%cladding_diameter: fiber cladding external diameter.

%ncore: refractive index of the core.

%n: refractive index of the external medium.

%r_eta: vector of domain points in the lateral direction.

%z_eta: vector of domain points in the axial direction.

%source: matrix modeling the extended source.

%OUTPUT VARIABLES

%eta_pointsource: stack of the collection efficiency with poin-like source.

%eta_convolved: stack of the collection efficiency with extended source.

%% Generation of the simmetry half-planes (implements eq. 3)

nclad=sqrt(ncore^2-NA^2);

NAeq=sqrt(nclad^2-n^2);

eta_core=maps_eta_halfplane(NA,core_diameter,n,r_eta,z_eta,0);

eta_clad=maps_eta_halfplane(NAeq,cladding_diameter,n,r_eta,z_eta,0)-maps_eta_halfplane(NAeq,core_diameter,n,r_eta,z_eta,0);

eta_halfplane=eta_core+eta_clad;

%% Volume reconstruction by rotation of the simmetry plane

[Z,R]=size(eta_halfplane);

eta_pointsource=zeros(2*R-1,2*R-1,Z);

[i1,i2,i3]=ndgrid(1:2*R-1,1:2*R-1,1:Z);

x=i1-R;

y=i2-R;

z=i3-1;

[~,r_out,z_out]=cart2pol(x,y,z);

[j1,j2]=meshgrid(1:R,1:Z);

r_in=j1-1;

z_in=j2-1;

eta_pointsource(:)=griddata(r_in,z_in,eta_halfplane,r_out(:),z_out(:),'linear');

eta_pointsource(isnan(eta_pointsource))=0;

%% Convolution between source and collection efficiency

eta_convolved=convn(eta_pointsource,source,'same');

eta_convolved(isnan(eta_convolved))=0;

end

function source=source(lateral,axial,domain)

% source models the microscope PSF as a gaussian fluorescent spot

% INPUT PARAMETERS

% lateral: FWHM of the PSF in the lateral dimensions [um]

% axial: FWHM of the PSF in the axial dimension [um]

% domain: axis of the domain in which the source should be represented [um]

% OUTPUT VARIABLES

% source: representation of the PSF

sigmasource_x=lateral/(2*sqrt(2*log(2)));

sigmasource_y=axial/(2*sqrt(2*log(2)));

sigmasource_z=lateral/(2*sqrt(2*log(2)));

[xmat_source,ymat_source,zmat_source]=meshgrid(domain,domain,domain);

source=exp(-(xmat_source.^2/(2*sigmasource_x^2)+ymat_source.^2/(2*sigmasource_y^2)+zmat_source.^2/(2*sigmasource_z^2)));

source=source/(sum(reshape(source,[],1)));

end

**Supplementary Script 2: data processing to obtain collection fields in quasi-transparent medium**

%% Processing of stack acquired in fluorescein

%% User-defined variables

% Fiber parameters

NA=0.50; %Numerical aperture

core_diameter=200; %Core diameter [?m]

cladding_diameter=225; %Cladding diameter [?m]

ncore=1.4613; %Core refractive index

% Medium parameters

n=1.335; %Medium refractive index

% Dataset files

filename_gain='gain_00001_00001.tif'; %Image for gain measurement

filename_stack='stack_00001_00001.tif'; %Volumetric stack

% Measurement parameters

z_focus=6; %Slice # at which the focal plane contains the optical axis

x_step=2.7; %Pixel size in the lateral direction [um]

z_step=5; %Pixel size in the axial direction [um]

rotation=0; %Rotation of the image [degree]

axis_index=244; %Pixel # individuating the optical axis

core_index=42; %Pixel # individuating the core facet

discard=0; %Number of pixel to discard due to rotation (zero padding)

n_of_slice=80; %Number of slice in the stack

n_of_frame=5; %Number of frame acquired for each slice

image_size=512; %Number of pixel of each slice side

photon_top_022=109.7259; %Normalization parameters for fluorescence excitation

% Analytical calculation parameters

r_an_step=5; %Step size along the lateral direction [?m]

z_an_step=5; %Step size along the axial direction [?m]

r_an_max=500; %Domain size along the lateral direction [?m]

z_an_max=1200; %Domain size along the lateral direction [?m]

r_an=[0:r_an_step:r_an_max];

z_an=[0:z_an_step:z_an_max];

r_an_full=horzcat(-fliplr(r_an(2:end)),r_an);

% Simulation parameters

grid_step_rt=25; %Step size of the ray tracing grid [?m]

r_rt=[0:19]*grid_step_rt;

z_rt=[0:60]*grid_step_rt;

% Source definition

source_lateral=3; %Lateral extension of the microscope PSF [?m]

source_axial=32; %Axial extension of the microscope PSF [?m]

source_domain=[-50:5:50]; %Side of the domain for source representation [?m]

% Processing parameters

smooth_factor_3d=3; %Size of the smoothing applied to the stack

smooth_factor_2d=3; %Size of the smoothing applied to the ray tracing map

smooth3d_enable=0; %If equal to 1, smoothing of the stack is enabled

smooth2d_enable=0; %If equal to 1, smoothing of the ray tracing is enabled

distance_mean_profile=30; %Number of pixel to average for profile normalization

% Plots parameters

longitudinal_step=50; %Step for the longitudinal planes visualization [?m]

transversal_step=100; %Step for the transversal planes visualization [?m]

%% END OF USER-DEFINED SECTION

% Do not edit below unless you really want to.

%% Load data (if not already in the workspace)

if ~exist('bench','var')

display('Loading data');

[gain_top,gain_bench,photon_top]=read_gain(filename_gain); %Calculates the gain of both acquisition channels and acquires the mean number of photon acquired from the microscope PMT

[top,bench,z,x,y]=read_stack(filename_stack,n_of_slice,n_of_frame,image_size,image_size,z_step,x_step,x_step,1,1); %Reads the stack acquired from both channels

top(top>max(reshape(top(1:512-core_index,:,:),1,[])))=0; %Removes outliers in the volume occupied by the fiber

import_rt; %Reads data from ray tracing simulations in transparent medium

import_rtb; %Reads data from ray tracing simulations in turbid medium

RT(:,3)=[]; %Removes unnecessary data

RT_brain(:,3)=[]; %Removes unnecessary data

end

%% Pre-processing of data: baseline removal, smoothing, and rotation

display('Data pre-processing');

bench_positive=bench-min(reshape(bench,1,[])); %Removes baseline

bench_smooth=bench_positive;

top_smooth=top;

if(smooth3d_enable==1) %Smoothing of data (if enabled)

bench_smooth=smooth3(bench_positive,'box',smooth_factor_3d);

top_smooth=smooth3(top,'box',smooth_factor_3d);

end

bench_smooth=flip(imrotate(bench_smooth,rotation,'bilinear'),1); %Rotates images

top_smooth=flip(imrotate(top_smooth,rotation,'bilinear'),1);

%% PMT count to photon number conversion and flat-field correction

top_normalized=normalize(top_smooth); %Normalizes stack acquired from the top PMT

bench_photon=(photon_top_022/photon_top)*bench_smooth./(gain_bench*top_normalized); %Converts the stack acquired from the fiber PMT in number of photons

bench_photon(bench_photon==Inf)=0; %Removes outliers

%% Errors determination

sigma_r_bench=sqrt(bench_smooth)./bench_smooth;

sigma_r_top=sqrt(top_smooth)./top_smooth;

sigma=sqrt(sigma_r_bench.^2+sigma_r_top.^2).*bench_photon;

%% Definition of the central slice for data analysis of measurement

eta_measure=bench_photon(:,:,z_focus);

%% Definition of the axial profile and integral on measurement

eta_profile=eta_measure(core_index:end-discard,axis_index);

%% Definition of analytical data (if not already in the workspace)

if ~exist('eta_pointsource','var')

display('Computation of analytical data');

nclad=sqrt(ncore^2-NA^2); %Evaluates cladding refractive index

NAeq=sqrt(nclad^2-n^2); %Evaluate equivalent numerical aperture at the interface cladding/medium

source=source(source_lateral,source_axial,source_domain); %Defines the fluorescent spot

[eta_pointsource,eta_convolved]=volume_eta(NA,core_diameter,cladding_diameter,ncore,n,r_an,z_an,source); %Evaluates the analytical collection efficiency

eta_pointsource=permute(eta_pointsource,[3 1 2]);

eta_convolved=permute(eta_convolved,[3 1 2]);

source=permute(source,[3 1 2]);

end

%% Definition of numerical data

display('Processing of numerical data');

eta_rt=RT_to_eta(RT,length(z_rt),grid_step_rt,grid_step_rt,0);

eta_rt_brain=RT_to_eta(RT_brain,length(z_rt),grid_step_rt,grid_step_rt,0);

eta_rt=flipud(eta_rt);

eta_rt_brain=flipud(eta_rt_brain);

if(smooth2d_enable==1) %Smoothing of data (if enabled)

eta_rt=filter2((1/smooth_factor_2d)^2*ones(smooth_factor_2d,smooth_factor_2d),eta_rt);

end

%% Evaluation of normalized collection volumes (if not already in the workspace)

volumes=[10 20 40 60 80]; %Percentages of maximum at which volumes should be evaluated

if ~exist('volumes_measurement','var')

display('Computation of measured and analytical collection volumes');

temp=cat(3,bench_photon(:,:,end:-1:z_focus),bench_photon(:,:,z_focus+1:end)); %Concatenates the stack with a mirrored version of itself to obtain a full volume

[xx,~,zz]=size(temp);

[~,volumes_measurement]=eval_isosurfaces([0:xx-1]*x_step,[0:xx-1]*x_step,[0:zz-1]*z_step,temp,3); %

clear xx zz temp;

end

%% Evaluation of cumulative number of photons (if not already in the workspace)

if ~exist('cumulative_photons','var')

temp=cat(3,bench_photon(:,:,end:-1:z_focus+1),bench_photon(:,:,z_focus:end)); %Concatenates the stack with a mirrored version of itself to obtain a full volume

temp2=cat(3,sigma(:,:,end:-1:z_focus+1),sigma(:,:,z_focus:end));

[~,~,z_center]=size(bench_photon(:,:,z_focus:end));

[cumulative_photons,plane_photons]=count_photons_in_volume(x_step,z_step,core_index,axis_index,z_center,temp,temp2);

clear temp temp2;

end

%% Plots

% Longitudinal planes (y=const)

figure(1);

for i=1:6

subplot(3,6,i);

imagesc(r_an_full,z_an,squeeze(eta_pointsource(:,:,length(r_an)+(i-1)*round(longitudinal_step/z_an_step))),[0 max(reshape(eta_pointsource,[],1))]);

axis equal;

axis([-max(r_an) max(r_an) min(z_an) max(z_an)]);

title(strcat('y=',num2str(r_an_full(length(r_an)+(i-1)*round(longitudinal_step/z_an_step))),'\mum'));

subplot(3,6,6+i);

imagesc(r_an_full,z_an,squeeze(eta_convolved(:,:,length(r_an)+(i-1)*round(longitudinal_step/z_an_step))),[0 max(reshape(eta_convolved,[],1))]);

axis equal;

axis([-max(r_an) max(r_an) min(z_an) max(z_an)]);

title(strcat('y=',num2str(r_an_full(length(r_an)+(i-1)*round(longitudinal_step/z_an_step))),'\mum'));

subplot(3,6,12+i);

temp=bench_photon(:,:,z_focus+(i-1)*round(longitudinal_step/z_step));

[xx,~]=size(temp);

imagesc(([0:xx-1]-axis_index)*x_step,([0:xx-1]-core_index)*x_step,temp,[0 max(reshape(bench_photon(:,:,z_focus),[],1))]);

axis equal;

axis([-max(r_an) max(r_an) min(z_an) max(z_an)]);

title(strcat('y=',num2str(z(z_focus+(i-1)*round(longitudinal_step/z_step))-z(z_focus)),'\mum'));

end

savefig('Figures/longitudinal_planes.fig');

% Transversal planes (z=const)

figure(2);

for i=1:6

subplot(3,6,i);

imagesc(r_an_full,r_an_full,squeeze(eta_pointsource(1+(i-1)*round(transversal_step/r_an_step),:,:)),[0 max(reshape(eta_pointsource,[],1))]);

axis equal;

axis([-max(r_an) max(r_an) -max(r_an) max(r_an)]);

title(strcat('z=',num2str(z_an(1+(i-1)*round(transversal_step/r_an_step))),'\mum'));

subplot(3,6,6+i);

imagesc(r_an_full,r_an_full,squeeze(eta_convolved(1+(i-1)*round(transversal_step/r_an_step),:,:)),[0 max(reshape(eta_convolved,[],1))]);

axis equal;

axis([-max(r_an) max(r_an) -max(r_an) max(r_an)]);

title(strcat('z=',num2str(z_an(1+(i-1)*round(transversal_step/r_an_step))),'\mum'));

subplot(3,6,12+i);

temp=flipud(squeeze(bench_photon(core_index+(i-1)*round(transversal_step/x_step),:,z_focus:end))');

[z_center,~]=size(temp);

temp=vertcat(temp,flipud(temp(2:end,:)));

[zz,xx]=size(temp);

imagesc(([0:xx-1]-axis_index)*x_step,([0:zz-1]-z_center)*z_step,temp,[0 max(reshape(bench_photon(:,:,z_focus),[],1))]);

axis equal;

axis([-max(r_an) max(r_an) -max(r_an) max(r_an)]);

title(strcat('z=',num2str(abs(x(core_index+(i-1)*round(transversal_step/x_step))-x(core_index))),'\mum'));

clear temp;

end

savefig('Figures/transversal_planes.fig');

% Plots normalized axial profiles

index_mean_profile=round(distance_mean_profile/x_step);

figure(3);

set(gca,'FontSize',20,'FontWeight','bold','LineWidth',2.5);

xlabel('Distance from the facet [\mum]','FontWeight','bold');

ylabel('Normalized axial profile [a.u.]','FontWeight','bold');

title(strcat('Axial profiles comparison for a',{' '},num2str(NA),'NA/',num2str(core_diameter),'\mum core fibers in fluorescein'));

hold on;

plot([0:length(eta_profile)-1]*x_step,eta_profile/mean(eta_profile(1:index_mean_profile)),'LineWidth',3);

plot(z_rt(2:end),eta_rt(2:end,length(r_rt))/max(eta_rt(:,length(r_rt))),'LineWidth',3);

plot(z_an,squeeze(eta_pointsource(:,length(r_an),length(r_an)))/max(eta_pointsource(:,length(r_an),length(r_an))),'LineWidth',3,'Color',[0.93 0.69 0.13]);

plot(z_an,squeeze(eta_convolved(:,length(r_an),length(r_an)))/max(eta_convolved(:,length(r_an),length(r_an))),'LineWidth',3,'Color',[0.49 0.18 0.56]);

legend('Experimental','Numerical','Analytical (point source)','Analytical (extended source)');

box();

grid();

axis([min(z_an) max(z_an) 0 1.2]);

hold off;

savefig('Figures/axial_profiles.fig');

% Plots normalized collection volumes

figure(4);

set(gca,'FontSize',20,'FontWeight','bold','LineWidth',2.5,'YScale','log');

xlabel('% of maximum number of photons','FontWeight','bold');

ylabel('Volume [\mum^3]','FontWeight','bold');

title(strcat('Collection volumes for a',{' '},num2str(NA),'NA/',num2str(core_diameter),'\mum core fibers in fluorescein'));

hold on;

plot(volumes,volumes_measurement,'LineWidth',3);

legend('Experimental','Analytical (point source)','Analytical (extended source)');

box();

grid();

axis([0 100 1e5 1e9]);

hold off;

savefig('Figures/normalized_volumes.fig');

% Plots cumulative number of photons

figure(5);

set(gca,'FontSize',20,'FontWeight','bold','LineWidth',2.5);

xlabel('z [\mum]','FontWeight','bold');

ylabel('Collected photons per plane (cumulative)','FontWeight','bold');

title(strcat('Photons collected from a',{' '},num2str(NA),'NA/',num2str(core_diameter),'\mum core fibers in fluorescein'));

hold on;

plot([0:length(cumulative_photons)-1]*x_step,cumulative_photons,'LineWidth',3);

box();

grid();

hold off;

savefig('Figures/cumulative_photons.fig');

% Plot efficiency maps

figure(6);

[xx,zz]=size(eta_measure);

imagesc(([0:xx-1]-axis_index)*x_step,([0:zz-1]-core_index)*x_step,eta_measure);

axis equal;

axis([-max(r_an) max(r_an) min(z_an) max(z_an)]);

set(gca,'FontSize',20,'FontWeight','bold','LineWidth',2.5);

title('Experimental collection efficiency map');

xlabel('x [\mum]');

ylabel('z [\mum]');

savefig('Figures/experimental_map.fig');

figure(7);

imagesc(r_an_full,z_an,squeeze(eta_pointsource(:,:,length(r_an))));

axis equal;

axis([-max(r_an) max(r_an) min(z_an) max(z_an)]);

set(gca,'FontSize',20,'FontWeight','bold','LineWidth',2.5);

title('Analytical (point source) collection efficiency map');

xlabel('x [\mum]');

ylabel('z [\mum]');

savefig('Figures/pointsource_map.fig');

figure(8);

imagesc(r_an_full,z_an,squeeze(eta_convolved(:,:,length(r_an))));

axis equal;

axis([-max(r_an) max(r_an) min(z_an) max(z_an)]);

set(gca,'FontSize',20,'FontWeight','bold','LineWidth',2.5);

title('Analytical (extended source) collection efficiency map');

xlabel('x [\mum]');

ylabel('z [\mum]');

savefig('Figures/convolved_map.fig');

figure(9);

r_rt_full=[-fliplr(r_rt(2:end)) r_rt];

imagesc(r_rt_full,z_rt,eta_rt);

axis equal;

axis([-max(r_an) max(r_an) min(z_an) max(z_an)]);

set(gca,'FontSize',20,'FontWeight','bold','LineWidth',2.5);

title('Numerical collection efficiency map');

xlabel('x [\mum]');

ylabel('z [\mum]');

savefig('Figures/numerical_map.fig');

figure(10);

imagesc(r_rt_full,z_rt,eta_rt_brain);

axis equal;

axis([-max(r_an) max(r_an) min(z_an) max(z_an)]);

set(gca,'FontSize',20,'FontWeight','bold','LineWidth',2.5);

title('Numerical collection efficiency map (in brain)');

xlabel('x [\mum]');

ylabel('z [\mum]');

savefig('Figures/numerical_map_brain.fig');

% Plots axial profiles (analytical and numerical without normalization)

figure(11);

set(gca,'FontSize',20,'FontWeight','bold','LineWidth',2.5);

xlabel('Distance from the facet [\mum]','FontWeight','bold');

ylabel('Normalized axial profile [a.u.]','FontWeight','bold');

title(strcat('Axial profiles comparison for a',{' '},num2str(NA),'NA/',num2str(core_diameter),'\mum core fibers in fluorescein'));

hold on;

plot(z_an,squeeze(eta_pointsource(:,length(r_an),length(r_an))),'LineWidth',3);

plot(z_rt(2:end),eta_rt(2:end,length(r_rt)),'LineWidth',3);

plot(z_rt(2:end),eta_rt_brain(2:end,length(r_rt)),'LineWidth',3);

legend('Analytical (point source)','Numerical (homogeneous)','Numerical (turbid)');

box();

grid();

axis([min(z_an) max(z_an) 0 0.038]);

hold off;

savefig('Figures/axial_profiles_fig1.fig');

**Supplementary Script 3: data processing to obtain collection fields in brain slice**

%% Processing of images acquired in brain slice

%% User defined variables

axis_index=263; %Pixel number identifying central slice.

core_index=122; %Pixel number identifying core facet.

x_step=2.7; %Step in the lateral direction.

n_of_frame=60; %Number of frame averaged in the acquisition.

image_size=512; %Image size in pixel (square image is assumed).

volumes=[10 20 40 60 80]; %Percentage at which isosurface are determined.

smooth_size=1; %Smoothing window size for volume determination.

%% Processing

%Reads measurement data

if ~exist('bench','var')

[top,bench,x,y]=read_grab('grab_00001_00001.tif',n_of_frame,image_size,image_size,x_step,x_step,1,1);

bench=bench-min(reshape(bench,1,[]));

bench=filter2(fspecial('average',11),bench);

halfplane=bench';

halfplane=halfplane(core_index:end,1:axis_index);

halfplane=fliplr(halfplane);

end

%Reconstruction of 3D data from central slice (measurements)

if ~exist('volume_reconstructed','var')

temp=volume_reconstruction(halfplane);

volume_reconstructed=permute(temp,[3 1 2]);

clear temp;

end

%Volume calculation

temp=cat(3,volume_reconstructed(:,:,end:-1:axis_index),volume_reconstructed(:,:,axis_index+1:end));

[xx,yy,zz]=size(temp);

temp(temp>500)=0;

[~,volumes_measurement]=eval_isosurface([0:xx-1]*x_step,[0:yy-1]*x_step,[0:zz-1]*x_step,temp,smooth_size);

clear temp xx yy zz;

%Isosurface visualization

temp=volume_reconstructed(:,:,end:-1:axis_index);

[xx,yy,zz]=size(temp);

temp(temp>500)=0;

[~,~]=eval_isosurface([0:xx-1]*x_step,[0:yy-1]*x_step,[0:zz-1]*x_step,temp,smooth_size,0,1);

clear temp xx yy zz;

%Plot of volumes

figure(2);

plot(volumes,volumes_measurement);

**Supplementary Script 4: data processing to obtain photometry efficiency**

%% Processing of images acquired in brain slice to determine rho

%% User defined variables

axis_index=290; %Pixel number identifying central slice.

core_index=142; %Pixel number identifying core facet.

rotazione=4; %Image rotation (in degree)

volumes=[10 20 40 60 80]; %Percentage at which isosurface are determined.

smooth_size=1; %Smoothing window size for volume determination.

x_step=2.7; %Step in the lateral direction.

n_of_frame=60; %Number of frame averaged in the acquisition.

image_size=512; %Image size in pixel (square image is assumed).

%% Processing

%Reads measurement data

if ~exist('bench','var')

[top,bench,x,y]=read_grab('grab_00001_00001.tif',n_of_frame,image_size,image_size,x_step,x_step,1,1);

[pinhole,~,~,~]=read_grab('ph_100_00001_00001.tif',n_of_frame,image_size,image_size,x_step,x_step,1,1);

end

%Registers images

[optimizer,metric]=imregconfig('multimodal');

registered=imregister(pinhole,bench,'translation',optimizer,metric);

%Pixel-by-pixel product

efficiency=imrotate(bench/max(reshape(bench,1,[])).*registered/max(reshape(registered,1,[])),rotazione,'bilinear');

[n_of_pixel,~]=size(efficiency);

%Smoothing of data

if ~exist('halfplane','var')

halfplane=efficiency';

halfplane=filter2(fspecial('average',11),halfplane);

halfplane=halfplane(core_index:end,1:axis_index);

halfplane=fliplr(halfplane);

end

%Volume reconstruction

if ~exist('volume_reconstructed','var')

temp=volume_reconstruction(halfplane);

volume_reconstructed=permute(temp,[3 1 2]);

clear temp;

end

%Volumes determination and isosurfaces visualization

if ~exist('volumes_measurement','var')

temp=cat(3,volume_reconstructed(:,:,end:-1:axis_index),volume_reconstructed(:,:,axis_index+1:end));

[xx,yy,zz]=size(temp);

[~,volumes_measurement]=superficidilivello([0:xx-1]*x_step,[0:yy-1]*x_step,[0:zz-1]*x_step,temp,smooth_size);

clear temp xx yy zz;

temp=volume_reconstructed(:,:,end:-1:axis_index);

[xx,yy,zz]=size(temp);

[~,~]=superficidilivello([0:xx-1]*x_step,[0:yy-1]*x_step,[0:zz-1]*x_step,temp,smooth_size,0,1);

clear temp xx yy zz;

end

%% Plots of results

%Plots of image acquired through microscope and fiber PMT (2P excitation),

%through pinhole PMT (1P excitation), and registering of fiber and pinhole

%PMT channels

figure(2);

subplot(2,2,1);

imagesc(([1:n_of_pixel]-core_index)*2.7,([1:n_of_pixel]-axis_index)*2.7,imrotate(top,rotazione,'bilinear'));

axis equal;

axis([0 900 -400 400]);

subplot(2,2,2);

imagesc(([1:n_of_pixel]-core_index)*2.7,([1:n_of_pixel]-axis_index)*2.7,imrotate(bench,rotazione,'bilinear'));

axis equal;

axis([0 900 -400 400]);

subplot(2,2,3);

imagesc(([1:n_of_pixel]-core_index)*2.7,([1:n_of_pixel]-axis_index)*2.7,imrotate(pinhole,rotazione,'bilinear'));

axis equal;

axis([0 900 -400 400]);

subplot(2,2,4);

imagesc(([1:n_of_pixel]-core_index)*2.7,([1:n_of_pixel]-axis_index)*2.7,imrotate(registered,rotazione,'bilinear'));

axis equal;

axis([0 900 -400 400]);

%Plots efficiency map

figure(3);

imagesc(([1:n_of_pixel]-core_index)*2.7,([1:n_of_pixel]-axis_index)*2.7,efficiency);

axis equal;

axis([0 900 -400 400]);

%Plots volumes

figure(4);

plot(volumes,volumes_measurement);

**Supplementary script 5: other functions**

function [cumulative_photons,plane_photons,sigma_cumulative]=count_photons_in_volume(x_step,z_step,core_index,axis_index,focus_index,stack,sigma)

%count_photons_in_volume Evaluates the cumulative number of photons and its uncertainty from a

%volumetric stack in a 800x400x900 um^3 volume.

%INPUT PARAMETERS

%x_step: step size along lateral and axial direction.

%z_step: step size along transversal direction.

%core_index: pixel number identifying core facet.

%core_index: pixel number identifying fiber axis in the lateral direction.

%focus_index: pixel number identifying fiber axis in the transversal direction.

%stack: volumetric stack from the measurement.

%sigma: uncertainty on stack.

%OUTPUT VARIABLES

%cumulative_photons: vector of cumulative number of photons.

%plane_photons: vector of number of photons collected from single planes.

%sigma_cumulative: uncertainty on cumulative_photons.

n_focus=ceil_even(400/z_step);

n_axial=round(900/x_step);

n_lateral=ceil_even(800/x_step);

stack_reduced=stack(core_index:core_index+n_axial,axis_index-n_lateral/2:axis_index+n_lateral/2,focus_index-n_focus/2:focus_index+n_focus/2);

sigma_reduced=sigma(core_index:core_index+n_axial,axis_index-n_lateral/2:axis_index+n_lateral/2,focus_index-n_focus/2:focus_index+n_focus/2);

plane_photons=squeeze(sum(sum(stack_reduced,3),2));

cumulative_photons=cumsum(plane_photons);

sigma_plane=squeeze(sum(sum(sigma_reduced.^2,3),2));

sigma_cumulative=sqrt(cumsum(sigma_plane));

end

function [maximum,volumes]=eval_isosurfaces(x,y,z,stack,smooth_size,varargin)

%eval_isosurfaces traces the isosurfaces on a volumetric stack. Maximum

%value of the stack as well volumes enclosed within the isosurfaces at 10%,

%20%, 40%, 60% and 80% of the maximum are returned.

%INPUT PARAMETERS

%x: distance vector along lateral dimension x.

%y: distance vector along lateral dimension y.

%x: distance vector along axial dimension z.

%stack: volumetric stack.

%smooth_size: smoothing intensity (must be an odd integer)

%OPTIONAL INPUT PARAMETERS

%reverse: if equal to 1 inverts z axis visualization.

%enable_output: if equal to 1 enables the plot of outputs.

%ctrl: if equal to 1 enables the visualization of voxel employed to

%determine enclosed volumes for each isosurfaces

%OUTPUT VARIABLES

%maximum: maximum value in the stack.

%volumes: volumes enclosed within the isosurfaces at 10%,

%20%, 40%, 60% and 80% of the maximum

if length(varargin)>=1

reverse=varargin{1};

end

if length(varargin)>=2

enable_output=varargin{2};

end

if length(varargin)>=3

ctrl=varargin{3};

end

if(~exist('enable_output','var'))

enable_output=0;

end

stack=smooth3(stack,'box',smooth_size);

maximum=max(max(max(stack)));

if(enable_output==1)

display('Maximum number of photon: ');

display(maximum);

end

[X,Y,Z]=ndgrid(x,y,z);

dx=abs(x(2)-x(1));

dy=abs(y(2)-y(1));

dz=abs(z(2)-z(1));

s10=isosurface(X,Y,Z,stack,0.1*maximum);

vox10=double(stack>=0.1*maximum);

v10=sum(sum(sum(vox10)))*dx^2*dz;

if(enable_output==1)

display(['Volume at 10% of maximum (' num2str(maximum*0.1) ') [um^3]: ']);

display(v10);

end

s20=isosurface(X,Y,Z,stack,0.2*maximum);

vox20=double(stack>=0.2*maximum);

v20=sum(sum(sum(vox20)))*dx^2*dz;

if(enable_output==1)

display(['Volume at 20% of maximum (' num2str(maximum*0.2) ') [um^3]: ']);

display(v20);

end

s40=isosurface(X,Y,Z,stack,0.4*maximum);

vox40=double(stack>=0.4*maximum);

v40=sum(sum(sum(vox40)))*dx^2*dz;

if(enable_output==1)

display(['Volume at 40% of maximum (' num2str(maximum*0.4) ') [um^3]: ']);

display(v40);

end

s60=isosurface(X,Y,Z,stack,0.6*maximum);

vox60=double(stack>=0.6*maximum);

v60=sum(sum(sum(vox60)))*dx^2*dz;

if(enable_output==1)

display(['Volume at 60% of maximum (' num2str(maximum*0.6) ') [um^3]: ']);

display(v60);

end

s80=isosurface(X,Y,Z,stack,0.8*maximum);

vox80=double(stack>=0.8*maximum);

v80=sum(sum(sum(vox80)))*dx^2*dz;

if(enable_output==1)

display(['Volume at 80% of maximum (' num2str(maximum*0.8) ') [um^3]: ']);

display(v80);

end

volumes=[v10 v20 v40 v60 v80];

if(enable_output==1)

figure(1);

hold on;

p10=patch(s10);

p10.FaceColor='k';

p10.FaceAlpha=0.25;

p10.LineStyle='none';

p20=patch(s20);

p20.FaceColor='b';

p20.FaceAlpha=0.25;

p20.LineStyle='none';

p40=patch(s40);

p40.FaceColor='g';

p40.FaceAlpha=0.25;

p40.LineStyle='none';

p60=patch(s60);

p60.FaceColor='y';

p60.FaceAlpha=0.25;

p60.LineStyle='none';

p80=patch(s80);

p80.FaceColor='r';

p80.FaceAlpha=0.25;

p80.LineStyle='none';

if(exist('reverse','var') && reverse==1)

set(gca,'Zdir','reverse');

end

set(gca,'LineWidth',2.5);

set(gca,'Box','on');

set(gca,'BoxStyle','back');

set(gca,'XGrid','on');

set(gca,'YGrid','on');

set(gca,'ZGrid','on');

set(gca,'FontSize',20);

set(gca,'FontWeight','bold');

xlabel('x [\mum]');

ylabel('y [\mum]');

zlabel('z [\mum]');

title('Isosurfaces of collection diagram [% of maximum]');

axis equal;

view(75,15);

legend('10%','20%','40%','60%','80%');

mTextBox=annotation('textbox');

mTextBox.FontSize=16;

mTextBox.Position=[0 0.2 0.1 0.1];

mTextBox.LineStyle='none';

stringamax=strcat('Max # of photons=',num2str(maximum));

stringa10=strcat('V_{10}=',num2str(v10),'{\mu}m^{3}');

stringa20=strcat('V_{20}=',num2str(v20),'{\mu}m^{3}');

stringa40=strcat('V_{40}=',num2str(v40),'{\mu}m^{3}');

stringa60=strcat('V_{60}=',num2str(v60),'{\mu}m^{3}');

stringa80=strcat('V_{80}=',num2str(v80),'{\mu}m^{3}');

set(mTextBox,'String',{stringamax,stringa10,stringa20,stringa40,stringa60,stringa80});

if(exist('ctrl','var') && ctrl==1)

figure(2);

hold on;

pp10=patch(isosurface(Y,X,Z,(double(vox10)),0.9));

pp20=patch(isosurface(Y,X,Z,(double(vox20)),0.9));

pp40=patch(isosurface(Y,X,Z,(double(vox40)),0.9));

pp60=patch(isosurface(Y,X,Z,(double(vox60)),0.9));

pp80=patch(isosurface(Y,X,Z,(double(vox80)),0.9));

pp10.FaceColor='k';

pp10.FaceAlpha=0.25;

pp10.LineStyle='none';

pp20.FaceColor='b';

pp20.FaceAlpha=0.25;

pp20.LineStyle='none';

pp40.FaceColor='g';

pp40.FaceAlpha=0.25;

pp40.LineStyle='none';

pp60.FaceColor='y';

pp60.FaceAlpha=0.25;

pp60.LineStyle='none';

pp80.FaceColor='r';

pp80.FaceAlpha=0.25;

pp80.LineStyle='none';

if(exist('reverse','var') && reverse==1)

set(gca,'Zdir','reverse');

end

axis equal;

view(75,15);

end

end

end

function output=normalize(input)

%normalize the input matrix between 0 and 1, returning it as output.

%INPUT PARAMETERS

%input: matrix to be normalized.

%OUTPUT VARIABLES

%output: normalized matrix.

output=(input-min(reshape(input,[],1)))/(max(reshape(input,[],1))-min(reshape(input,[],1)));

end

function eta=RT_to_eta(matrix,z_length,z_step,r_step,visualize)

%RT_to_eta(matrix,z_length,z_step,y_step) create the matrix of collection

%efficiency from the output of Zemax.

%INPUT PARAMETERS

%matrix: Nx3 matrix containing y (1st column), z (2nd column) and power

%(3rd column) from Zemax.

%z: number of simulation points along the fiber axis.

%z_step: grid step along the fiber axis (in meters).

%r_step: grid step along the radial axis (in meters).

%OUTPUT VARIABLES

%eta: 2D map of collection efficiency.

[n_rows,~]=size(matrix);

r_length=n_rows/z_length;

eta=reshape(matrix(:,3),r_length,z_length)';

eta=horzcat(fliplr(eta(:,2:end)),eta);

if visualize==1

[zl,rl]=size(eta);

figure(1);

imagesc([-(rl-1)/2:(rl-1)/2]*r_step*1e3,[zl-1:-1:0]*z_step*1e3,eta);

set(gca,'FontSize',20,'FontWeight','bold','LineWidth',2.5);

colorbar('LineWidth',2.5);

xlabel('r [mm]','FontWeight','bold');

ylabel('z [mm]','FontWeight','bold');

title('Collection efficiency');

axis equal;

axis([-0.4 0.4 0 1.2]);

end

end

function [volume]=volume_reconstruction(halfplane)

%% Volume reconstruction from the simmetry plane

[Z,R]=size(halfplane);

volume=zeros(2*R-1,2*R-1,Z);

[i1,i2,i3]=ndgrid(1:2*R-1,1:2*R-1,1:Z);

x=i1-R;

y=i2-R;

z=i3-1;

[~,r_out,z_out]=cart2pol(x,y,z);

[j1,j2]=meshgrid(1:R,1:Z);

r_in=j1-1;

z_in=j2-1;

volume(:)=griddata(r_in,z_in,halfplane,r_out(:),z_out(:),'linear');

volume(isnan(volume))=0;

end

function [top,bench,x,y]=read_grab(filename,n_of_frame,image_size_x,image_size_y,x_step,y_step,gain_top,gain_bench)

%read_grab imports a two-channel grab acquired with ScanImage into Matlab

%workspace. At the same time, it creates two tiff files with the same data,

%one per each channel.

%INPUT PARAMETERS

%filename: name of the file to be read, with extension.

%n_of_frame: number of acquired frame to be averaged.

%image_size_x: dimension, in pixel, of the image along axis x.

%image_size_y: dimension, in pixel, of the image along axis y.

%x_step: spatial sampling step along x.

%y_step: spatial sampling step along y.

%gain_top: gain of channel 1.

%gain_bench: gain of channel 2.

%OUTPUT VARIABLES

%top: image_size x image_size matrix with data from channel 1 (average on

%n_of_frame frames).

%bench: image_size x image_size matrix with data from channel 2 (average on

%n_of_frame frames).

%x: vector containing spatial axis along x.

%y: vector containing spatial axis along y.

%OUTPUT FILES

%top.tiff: image with grab acquired on channel 1.

%bench.tiff: image with grab acquired on channel 2.

%Definition of spatial output variables

x=[0:image_size_x-1]*x_step;

y=[0:image_size_y-1]*y_step;

%Reads the file containing the grab

grab=ScanImageTiffReader(filename).data();

%Splits the two channels and determines frame average and converts in

%number of photons

top=double(median(grab(:,:,1:2:2*n_of_frame-1),3)/gain_top); %odd frames

bench=double(median(grab(:,:,2:2:2*n_of_frame),3)/gain_bench); %even frames

%Converts data to uint16 for file writing.

top16=uint16(top);

bench16=uint16(bench);

%Writes the output files.

imwrite(top16,'top.tiff');

imwrite(bench16,'bench.tiff');

end

function [top,bench,z,x,y]=read_stack(filename,n_of_slice,n_of_frame,image_size_x,image_size_y,z_step,x_step,y_step,gain_top,gain_bench)

%read_stack imports a two-channel stack acquired with ScanImage into Matlab

%workspace. At the same time, it creates two tiff files with the same data,

%one per each channel.

%INPUT PARAMETERS

%filename: name of the file to be read, with extension.

%n_of_slice: number of slices acquired in the stack.

%n_of_frame: number of acquired frame to be averaged.

%image_size_x: dimension, in pixel, of the image along axis x.

%image_size_y: dimension, in pixel, of the image along axis y.

%z_step: spatial sampling step along z.

%x_step: spatial sampling step along x.

%y_step: spatial sampling step along y.

%gain_top: gain of channel 1.

%gain_bench: gain of channel 2.

%OUTPUT VARIABLES

%top: image_size x image_size matrix with data from channel 1 (average on

%n_of_frame frames).

%bench: image_size x image_size matrix with data from channel 2 (average on

%n_of_frame frames).

%z: vector containing spatial axis along z.

%x: vector containing spatial axis along x.

%y: vector containing spatial axis along y.

%OUTPUT FILES

%top.tiff: image with grab acquired on channel 1.

%bench.tiff: image with grab acquired on channel 2.

%Definition of spatial output variables

x=[0:image_size_x-1]*x_step;

y=[0:image_size_y-1]*y_step;

z=[0:n_of_slice-1]*z_step;

%Reads the file containing the grab

stack=double(ScanImageTiffReader(filename).data());

%Splits the two channels and converts in number of photons

top_temp=stack(:,:,1:2:2*n_of_slice*n_of_frame-1)/gain_top; %odd frames

bench_temp=stack(:,:,2:2:2*n_of_slice*n_of_frame)/gain_bench; %even frames

%Reshapes the matrices for frame average in each slice

top_reshape=reshape(top_temp,image_size_x,image_size_y,n_of_frame,n_of_slice);

bench_reshape=reshape(bench_temp,image_size_x,image_size_y,n_of_frame,n_of_slice);

%Calculates frame average in each slice

top=squeeze(mean(top_reshape,3));

bench=squeeze(mean(bench_reshape,3));

%Converts data to uint16 for file writing.

top16=uint16(top);

bench16=uint16(bench);

%Writes the output files.

delete('topstack.tiff','benchstack.tiff');

for i=1:n_of_slice

imwrite(top16(:,:,i),'topstack.tiff','writemode','append');

imwrite(bench16(:,:,i),'benchstack.tiff','writemode','append');

end

end

function [gain_top,gain_bench,photon_top]=read_gain(filename)

%read_gain determines the gain on two acquisition channels based on Poisson

%statistics. The two channels are acquired in the same file through

%ScanImage.

%INPUT PARAMETERS

%filename: name of the file to be read, with extension.

%OUTPUT VARIABLES

%gain_top: gain of channel 1.

%gain_bench: gain of channel 2.

top=reshape(double(imread(filename,1)),1,[]);

bench=reshape(double(imread(filename,2)),1,[]);

gain_top=var(top)/mean(top);

gain_bench=var(bench)/mean(bench);

photon_top=mean(top)/gain_top;

end
